# Bedaquiline, pretomanid and linezolid for treatment of extensively drug resistant, intolerant or non-responsive multidrug resistant pulmonary tuberculosis

**DOI:** 10.1056/NEJMoa1901814

**Published:** 2020-03-05

**Authors:** Francesca Conradie, Andreas H Diacon, Nosipho Ngubane, Pauline Howell, Daniel Everitt, Angela M Crook, Carl M Mendel, Erica Egizi, Joanna Moreira, Juliano Timm, Timothy D McHugh, Genevieve Wills, Anna Bateson, Robert Hunt, Christo Van Niekerk, Mengchun Li, Morounfolu Olugbosi, Melvin Spigelman

**Affiliations:** 1 Clinical HIV Research Unit, Faculty of Health Sciences, University of Witwatersrand, South Africa; 2 Sizwe Tropical Disease Hospital, Sandringham, South Africa; 3 Task Applied Science and Stellenbosch University, Cape Town, South Africa; 4 King DiniZulu Hospital Complex, Durban, South Africa; 5 Global Alliance for TB Drug Development, New York, USA; 6 Institute of Clinical Trials and Methodology, University College London, UK; 7 UCL Centre for Clinical Microbiology, University College London, UK; 8 Global Alliance for TB Drug Development, Pretoria, South Africa

## Abstract

**Background:**

Patients with extensively drug resistant tuberculosis (TB) have limited treatment options with historically poor outcomes. We investigated treatment with 3 oral drugs, bedaquiline (B), pretomanid (Pa) and linezolid (L), (B-Pa-L), with TB bactericidal activity and little pre-existing resistance.

**Methods:**

Nix-TB is an open label single arm study ongoing at three South African sites evaluating the safety and efficacy of B-Pa-L for 26 weeks for extensively drug-resistant TB or treatment intolerant /non-responsive multidrug-resistant TB. We present the efficacy and safety outcomes for all 109 patients enrolled in the trial followed to the predefined primary endpoint, six months after the end of treatment.

**Results:**

In the intent to treat analysis, 98 patients (90%), (95% CI 82.7-94.9%) had a favourable outcome at 6 months after the end of treatment. Six patients died during the early stages of treatment, one withdrew during treatment, one died during follow-up without evidence of relapse, one relapsed, one relapsed and subsequently died during follow up and one was lost to follow-up. The expected linezolid toxicities of peripheral neuropathy (experienced by 81% of patients) and myelosuppression (48%), while common, were manageable, often requiring reductions of dose and/or interruptions in linezolid.

**Conclusions:**

These results suggest that B-Pa-L is a viable option for tuberculosis patients with highly resistant forms of TB, provided adequate safety management is available.

*Trial registration*: ClinicalTrials.gov Identifier: NCT02333799

*Sponsor*: Global Alliance for TB Drug Development (TB Alliance)

## Background

The vision of the End TB strategy of the World Health Organization (WHO) is for a world free of tuberculosis (TB) by the year 2035.[1] A potential threat to this is the lack of effective treatment for intolerant or non-responsive multi-drug resistant TB (MDR-TB, with resistance to isoniazid and rifampicin) or extensively drug-resistant TB (XDR-TB, MDR-TB with additional resistance to any fluoroquinolone and at least 1 injectable (amikacin, capreomycin, or kanamycin)).[2]

The treatment of MDR-TB, at the time of starting the trial ranged from 18 months to over 2 years, with some patients receiving up to 7 medications including a second-line injectable. Intolerance to these regimens is high with 45% of patients having moderate to severe adverse events.[3] XDR-TB patients had few treatment options with no standard treatment regimen. The published successful treatment rate of XDR-TB across South Africa was low and consistent, averaging 14%, with a range of 2% to 22%.[4, 5]

Bedaquiline (B) is a diarylquinoline that inhibits the mycobacterial adenosine triphosphate (ATP) synthase.[6] A Phase 2 study of bedaquiline added to a background regimen reported that 23 of 38 patients (61%) with XDR-TB were responders at 120 weeks after initiation of treatment.[7] There has been increased early access to this medication, especially in South Africa. Among the cohort of XDR-TB patients initiating treatment between July 2014 to March 2016, bedaquiline-containing regimens were associated with a reduction in the risk of all-cause mortality (hazard ratio [HR] 0.26, 95% CI 0.18–0.38) compared with regimens not containing bedaquiline.[8]

Linezolid (L), an oxazolidinone, approved in many countries for drug-resistant, gram-positive bacterial infections, inhibits bacterial protein synthesis.[9] Resistance of MTB to linezolid is rare as this drug has not been widely used to treat TB. A recent evaluation of 420 XDR and MDR TB strains in S. Korea found only one (0.3 %) with resistance at the WHO recommended cut off.[10]

Pretomanid (Pa), a nitroimidazooxazine inhibits mycolic acid biosynthesis and thereby blocks mycobacterial cell wall production also acts as a respiratory poison against non-replicating bacteria following nitric oxide release under anaerobic conditions.[11, 12] Pretomanid demonstrates *in vitro* activity against both drug-susceptible and drug-resistant (including XDR) strains of *Mycobacterium tuberculosis* (MTB) and has *in vivo* activity in animal models of TB. [13, 14] Phase 2 studies to evaluate the early bactericidal activity (EBA) of pretomanid over 14 days of daily oral monotherapy showed that the lowest dose to produce a maximal EBA effect was 100 mg/day.[15] Pretomanid was recently approved by the US Food and Drug Administration (FDA) under the Limited Population Pathway for Antibacterial and Antifungal Drugs (LPAD) as part of a combination regimen with bedaquiline and linezolid for the treatment of adults with pulmonary extensively drug resistant (XDR), treatment-intolerant or nonresponsive multidrug-resistant (MDR) tuberculosis (TB). Here we present the results of the Nix-TB trial which evaluated the safety, tolerability, efficacy, and pharmacokinetics (PK) of this new oral regimen, B-Pa-L.

## Methods

### Study Design

Nix-TB is an open-label, single arm trial in patients with either XDR-TB or treatment intolerant/non-responsive (TI/NR) MDR-TB. All participants received 26 weeks of oral daily treatment with an option to extend treatment duration to 39 weeks in participants who were culture positive at week 16. An interim analysis for safety was conducted every 15 patients.

### Study Participants

Participants aged 14 years and above were eligible for enrollment. The major inclusion criteria were: pulmonary XDR-TB or MDR-TB documented on culture or molecular test within 3 months prior to screening with drug resistance documented by phenotypic or genotypic tests followed by, in the MDR-TB cases, documented non-response to treatment with an available regimen for 6 months or more prior to enrolment, or inability to continue a second-line drug regimen due to documented drug intolerance. HIV-infected participants having a CD4+ count > 50 cells/μL could be enrolled and appropriate antiretroviral treatment given (See Supplementary material (S1)). Participants with baseline peripheral neuropathy of grade 3 or 4 were excluded. Detailed inclusion and exclusion criteria are given in the Protocol (Supplementary material). All participants provided written informed consent.

### Study Sites

Participants were enrolled from three study sites in South Africa: Sizwe Tropical Disease Hospital, Johannesburg, Task Applied Science at Brooklyn Chest Hospital, Cape Town and King DiniZulu Hospital Complex in Durban.

### Enrollment and intervention

Participants received orally administered treatment as follows:

- bedaquiline, 400 mg once daily for 2 weeks followed by, 200 mg 3 times a week for 24 weeks plus
- pretomanid 200 mg daily for 26 weeks plus
- linezolid 1200 mg daily for up to 26 weeks (with dosage adjustment depending on tolerability or toxicity)

The total daily dose of linezolid of 1200 mg was changed from 600 mg a twice daily to 1200 mg a once daily scheme during the trial to evaluate primarily whether a single daily dose, which would reduce the exposures above the potential threshold for mitochondrial protein synthesis toxicity, would have less clinical toxicity. More details can be found in the Protocol (Supplementary material).

### Study schedule

All participants were screened within 9 days before receiving the first dose of the trial treatment on Day 1 and were seen weekly thereafter to week 16, then at weeks 20 and 26, then post end of treatment, at months 1, 2, 3 and every 3 months thereafter to 24 months after the end of treatment.

### Microbiology

At screening and most subsequent visits, as scheduled, two sputum samples were collected for smear microscopy and culture using the Mycobacteria Growth Indicator Tube (MGIT) method and MTB was identified by molecular methods. MTB isolates at baseline and at end of treatment or during follow-up were transferred to a central laboratory for determination of: minimum inhibitory concentration (MIC) of bedaquiline, pretomanid, and linezolid; drug susceptibility testing in the MGIT system for rifampicin, isoniazid, streptomycin, ethambutol, moxifloxacin and kanamycin; and paired whole genome sequencing (WGS). The Laboratory Manual includes full details of the microbiological procedures (Supplementary material).

### Safety

Assessments included regular ECGs and blood draws with particular attention to expected haematological toxicities. Ophthalmological examination including visual acuity and color vision assessment was done every 4 weeks and a slit lamp examination for cataracts was done at screening, end of treatment and three months later. Changes were noted in signs and symptoms of peripheral neuropathy, including any changes from baseline, using a Brief Peripheral Neuropathy rating scale that evaluated subjective symptoms and objective measures of deep tendon reflexes and vibration sense. [16]

### Outcome measures and endpoints

The primary endpoint of unfavorable outcome was the incidence of bacteriologic failure, relapse or clinical failure or death through follow-up until 6 months after the end of treatment. Participants were considered to have a favourable outcome if their clinical TB disease resolved and they had a negative culture status at 6 months from end of therapy, and had not already been classified as having an unfavorable outcome. Secondary endpoints included: time to unfavorable outcome and time to sputum culture conversion through the treatment period. Culture conversion required at least 2 consecutive culture negative samples collected at least 7 days apart.

Safety and tolerability endpoints include all-cause mortality, incidence of treatment-emergent adverse events (TEAEs) occurring from the start of treatment. Here, we are reporting the TEAEs through 14 days after the end of treatment, categorised by grade according to the division of microbiology and infectious diseases (DMID) severity,[17] and drug relatedness and seriousness.

### Study oversight

An independent data safety monitoring committee (DSMC) oversaw the safety of the trial and advised to continue the trial without change after each review. While there were no formal stopping rules, recommendations for early stopping or modifying the trial was left to the discretion of the DSMC.

National and local ethics committees approved the study. The U.S. Food and Drug Administration and the Medicines Control Council in South Africa reviewed and approved the protocol.

## Statistical analysis

### Sample size

No formal statistical power calculation was performed; however, for trial success, the lower bound of the 95% confidence interval (CI) for a favourable outcome was pre-specified as being above 50%. While the protocol allowed enrolment of up to 200 participants, enrolment to Nix-TB stopped after 109 when ZeNix, a randomized trial to investigate optimal linezolid dosing and duration within the BPaL regimen, was started. All analyses were performed in Stata Version 15.1.

### Primary analysis

One hundred and nine patients were included in this analysis for both efficacy and safety. Two participants had their treatment extended to 9 months. Intent-to-treat (ITT), modified ITT (mITT), which was pre-specified as the primary analysis, and per-protocol (PP) analyses were performed. However, populations were similar and for ease of interpretation, here we report the most conservative analysis (i.e. ITT with no exclusions). More details of the analysis population definitions can be found in the Statistical Analysis Plan (Supplementary material).

The proportion with exact 95% confidence interval for the proportion of assessable patients with a favourable outcome, was calculated. Subgroup analyses of the primary outcome by TB type (XDR-TB, MDR-TB), HIV status and dosing schedule of linezolid were performed to evaluate the consistency of the results. No formal statistical tests were performed.

### Secondary outcomes

Time to unfavorable outcome and time to culture negative status were analysed using standard time-to-event analysis techniques including Kaplan Meier plots.

## Results

Forty three potential participants were screened out for ineligibility (see supplementary material S2) and 109 patients were enrolled in the trial between 16 April 2015 and 15 November 2017. The first 44 participants were started on linezolid 600 mg bid and the remaining 65 were started on 1200 mg qd. Two participants had their treatment extended beyond 6 months.

The demographic and clinical characteristics of the participants are given in Table 1. The median age was 35 years (range 17 to 60 years); 57 (52.3%) were male; 56 (51.4%) participants were HIV positive; 92 (84.4%) had cavities on chest X-ray and the median BMI was 19.7 kg/m^2^. The median time from diagnosis of current TB was 12 months (range <1 to 141 months). Seventy-one (65.1%) were classified as XDR-TB, 19 (17.4%) MDR (non-responsive) and 19 (17.4%) MDR-intolerant.

**Table 1:** Baseline characteristics of patients enrolled in Nix-TB

		Total
Total enrolled		**109**
Age (years)	Median	**35**
	Min-Max	**17-60**
Sex N (%)	Male	**57 (52.3)**
Race N (%)	Black	**83 (76.1)**
	Mixed race	**25 (22.9)**
	White	**1 (0.9)**
BMI (kg/m^2^)	Median	**19.7**
	Min-Max	**12.4-41.1**
HIV status N (%)	Positive	**56 (51.4)**
Time since HIV diagnosis (years)	Median	**4.0**
	Min-Max	**0.2-14.3**
CD4 count^a^ (cells/uL)	Median	**343**
	Min-Max	**55-1023**
Cavities present on	No	**17 (15.6)**
chest x-ray	Unilateral	**51 (46.8)**
	Bilateral	**41 (37.6)**
Karnosfsky Score N (%)	100 (no complaints)	**9 (8.3)**
	90	**50 (45.9)**
	80	**29 (26.6)**
	70	**19 (17.4)**
	60	**2 (1.8)**
	< 60	**0**
Duration since current TB diagnosis (months)	Median	**12**
	Min-Max	**<1 -141**

a 5 patients had missing CD4 count

### Microbiology

All participants met inclusion criteria with documented resistance to anti-TB drugs to categorize them as having infection with either XDR- or MDR-MTB organisms, although 16 did not have positive baseline cultures. Baseline isolates for 58 participants could be evaluated for MICs to all study drugs: except for 3 isolates, bedaquiline and linezolid MICs were all < 1 μg/mL, values below or equal to the critical concentrations recommended by WHO (1 μg/mL for both drugs). [18] Two baseline isolates had bedaquiline MICs equal to 2 and 1 equal to 4 μg/mL. All baseline isolates tested had pretomanid MIC ≤ 1 μg/mL.

### Efficacy analysis primary end point

The number of participants classified as favourable in the ITT (no exclusions) analysis was 98 (90%), (95% CI 82.7-94.9%), with similar findings for mITT and PP (Table 2). These results demonstrate a lower bound of the 95% confidence interval substantially greater than 50% (Table 2).

**Table 2:** Primary efficacy analysis ITT, mITT and PP analysis populations

	Total	XDR	TI/NR MDR
Total enrolled	**109**	71	38
ITT^a^	**109**	71	38
Favourable N (%)	**98 (90)**	63 (89)	35 (92)
95% Confidence Interval	**(82.7, 94.9)**	(79.0, 95.0)	(78.6,98.3)
Unfavourable N (%)	**11 (10)**	8 (11)	3 (8)
Deaths N	7	6	1
Withdrawal during treatment	1	1	0
Lost to follow-up (after end of treatment)	1	0	1
Relapse N	2^b^	1	1
mITT^a^	**107**	70	37
Favourable N (%)	**98 (92)**	63 (90)	35 (95)
95% Confidence Interval	**(84.6, 96.0)**	(80.4,95.8)	(81.8,99.3)
Unfavourable N (%)	**9 (8)**	7 (10)	2 (5)
Deaths N	6	5	1
Withdrawal during treatment	1	1	0
Relapse N	2^b^	1	1
PP	**105**	68	37
Favourable N (%)	**97 (92)**	62 (91)	35 (95)
95% Confidence Interval	**(85.5, 96.7)**	(81.8,96.7)	(81.8,99.3)
Unfavourable N (%)	**8 (8)**	6 (9)	2 (5)
Deaths N	6	5	1
Relapse N	2^b^	1	1

a Primary analysis population;

b 1 relapse did not have a baseline isolate available

Two exclusions in mITT, non-TB related death in follow-up; lost to follow-up after end of treatment. Two further exclusions from PP, 1 inadequate amount of drug; 1 withdrawn (not for treatment failure) during treatment.

The reasons for the 11 unfavorable outcome under -ITT were death during treatment (n=6), death from an unknown cause, but not considered TB or drug related during follow-up (n=1); total deaths=7; withdrawal of consent during treatment (n=1), relapse in follow-up (n=2) and loss to follow up during follow up (n=1).

### Sub-group analyses

The results were similar when stratified by TB type. For the 71 XDR-TB participants in ITT, the number classified as favourable was 63 (89%), (95% CI 79.0-95.0%) and for the 38 MDR patients, 35 (92%) were favourable (95% CI 78.6-98.3%), see Table 2. Results were also consistent across HIV status and linezolid dosing scheme.

**Figure 1:**
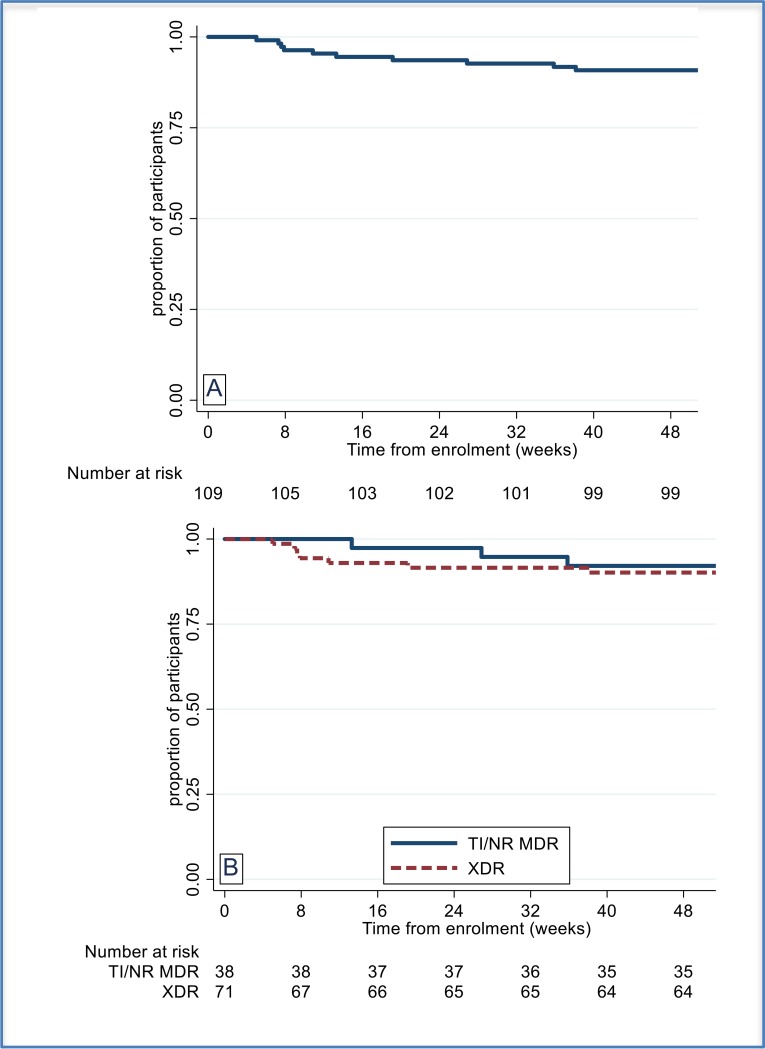
Time to unfavourable outcome (ITT) A Overall; B by TB Type

**Figure 2:**
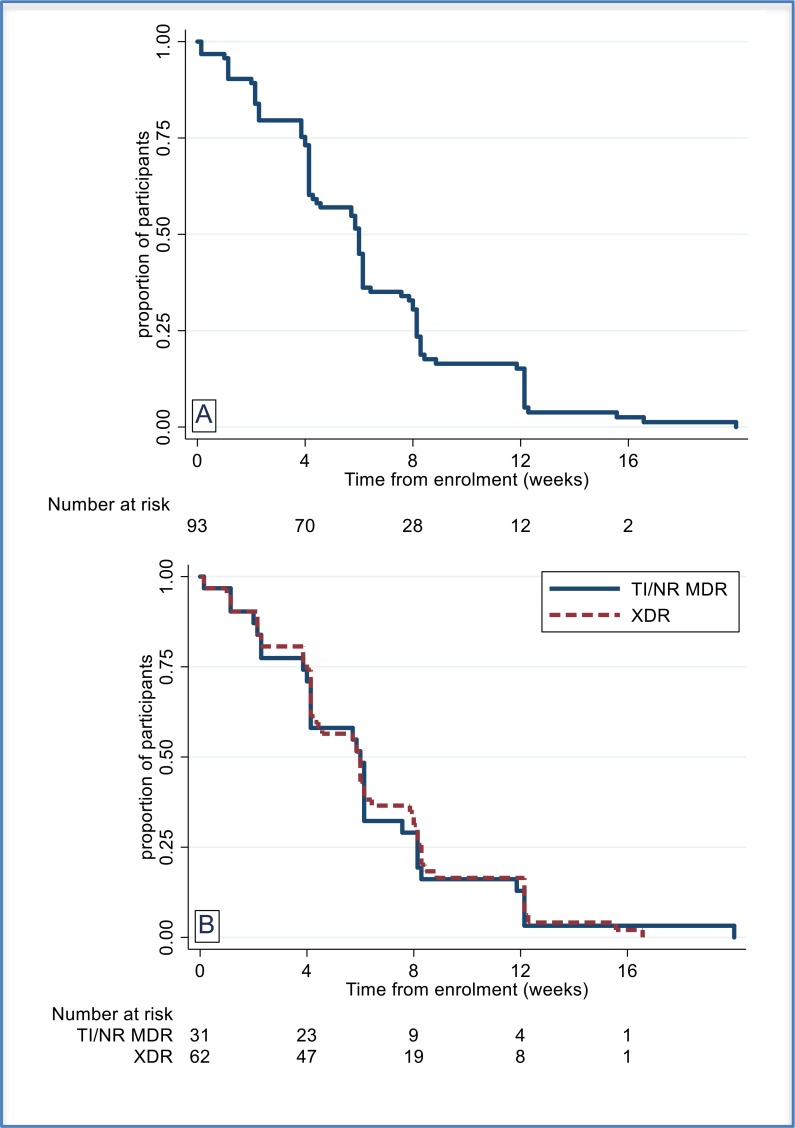
Time to culture negative status for those positive at baseline (ITT) A Overall; B by TB Type

Time to unfavorable outcome, overall and stratified by TB type, HIV status and linezolid dosing is shown in Figure 1. Kaplan Meier estimates of time to culture negative status, overall and stratified by TB type, are shown in Figure 2**.** There were two relapses. WGS performed for one on the corresponding baseline/late isolate pair confirmed the participant had a relapse with the same MTB organism (5 single nucleotide polymorphisms [SNPs] separate the 2 isolates). One of these SNPs produced a change in the bedaquiline resistance gene *Rv0678*, from wild type at baseline to a 138-139 insG variant in the late isolate. This patient had an elevated bedaquiline MIC in the late isolate (MIC = 4 μg/mL vs 0.5 μg/mL at baseline).[19]. The second participant with a relapse did not have a baseline isolate available for testing, but the late isolate was analysed and shown to be susceptible to all three of the study drugs.

### Safety analysis outcomes

All participants had at least one TEAE, and 19 (17.4%) had serious adverse events that were similar across HIV status. Six participants died during the course of treatment (at months 1 (n=1), 2 (n=4) and 3 (n=1); An additional participant died from sepsis and gangrene after relapse of TB. Sixty-two (57%) had TEAEs of grade 3 or more, similar by HIV status (See Table 3). More details on the adverse events and deaths are provided in the Supplementary material (S3 and S4).

**Table 3:** Treatment emergent adverse events (TEAE) by HIV status and linezolid regimen

	HIV status			Linezolid regimen	
	Total	Negative	Positive	600mg BID	1200mg QD
At least 1 treatment emergent adverse event (TEAE) N	**109**	53	56	44	65
^a^TEAE leading to death N (%)	**6 (5.5)**	3 (5.7)	3 (5.4)	4 (9.1)	2 (3.1)
^a^Serious TEAE N (%)	**19 (17.4)**	10 (18.9)	9 (16.1)	13 (29.5)	6 (9.2)
^a^Grade 3 or 4 TEAE N (%)	**62 (56.9)**	27 (50.9)	35 (62.5)	27 (61.4)	35 (53.8)

BID=Twice a day; QD=4 times per day;

a patients can appear in more than 1 row

**Figure 3:**
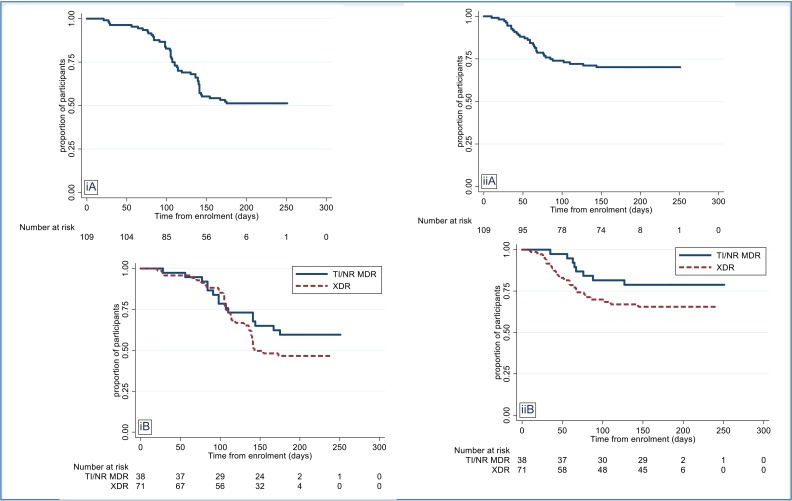
Time to first reduction or interruption of linezolid i) Due to Peripheral neuropathy; ii) Due to Myelosuppression; A Overall; by TB Type;

Eighty-eight (81%) participants had peripheral neuropathy reported on treatment, the majority of which were mild to moderate symptoms. Figure 3A shows the time to first linezolid dose reduction or interruption for neuropathy, with the majority of these occurring after the initial 3 months of treatment. Results were similar between those who were HIV co-infected or uninfected and between those initially receiving linezolid 600 mg bid vs 1200 qd. Two participants developed optic neuritis which resolved on withdrawal of linezolid. Blood and lymphatic system disorders were the second most common TEAE with 40 (37%) having TEAEs of anemia, seven of whom had hemoglobin decreases to less than 8.0 gm/dL. Figure 3B shows the time- to-first linezolid dose reduction or interruption for anemia. The majority of these were in the first 2 months of treatment, with similar results observed between HIV co-infected and uninfected participants and between the different starting doses of linezolid.

Twelve patients had transaminase increases; 12 had an ALT elevation and 11 an AST elevation greater than 3 times the upper limits of normal (ULN). Two of these had ALT and AST elevations of >3 × ULN as well as direct and total bilirubin elevations of >2 × the ULN. In both cases the study drug regimen was interrupted. Eight patients had the regimen interrupted for hepatic adverse events, but all resumed and completed the full 26 weeks of treatment. The maximum mean increase in the QT-interval by Fridericia’s method was 10 msec at Week 16; no participant had an increase > 480 msec.

All surviving participants completed 26 weeks (including two who extended to 39 weeks) of treatment with allowable interruptions of up to 35 consecutive days, and none had the regimen permanently discontinued. Most participants required a reduction in dose or interruption of linezolid during treatment. 37 (34%) participants completed 26 weeks of linezolid without any interruption, although they may have had a dose reduction, and 16 (15%) completed 26 weeks at a 1200 mg total daily dose of linezolid with no interruptions or dose reductions.

## Discussion

A favourable outcome 6 months after the completion of therapy was found in 98 patients (90%), including 63 (89%) XDR-TB patients. Thirty-eight patients enrolled in the Nix-TB Trial had MDR-TB, with 35 (92%) favourable outcomes.

One of the limitations of this study is that there was no randomized control group. At the time of implementing this protocol, there was no standard regimen for the treatment of XDR-TB. Mortality was extremely high, with long term cure below 20% in reports from South Africa, and a single arm trial was therefore warranted. However, during the course of this study both bedaquiline and linezolid have been increasingly used to treat MDR and XDR-TB , and the WHO recently published guidelines that recommend these 2 drugs be used first line to treat patients with MDR-TB over an 18 month course of therapy.[20] Bedaquiline has been shown recently in a programmatic setting to reduce overall mortality when added to treatment for MDR- and XDR-TB.[8, 21, 22] Patients with XDR-TB treated in Cape Town, South Africa at one of the sites of the Nix-TB Trial have reported cure rates that were below 20% prior to the use of bedaquiline or linezolid and that have improved to a rate of 66% more recently when bedaquiline and linezolid (81% of the 68 patients) were added to their regimen.[23] Of note, these patients were newly diagnosed and treated for 24 months with a median of 8 drugs.

Another limitation of this study was that it was conducted in only one country which potentially limits the generalizability of the findings. South Africa was selected as it has a robust regulatory framework, good clinical trial capacity and historically poor outcomes in XDR-TB. In addition, there is a high background prevalence of HIV infection.

The primary endpoint was based on treatment failure at 6 months after the end of treatment as most relapses occur in this period.[24] The secondary endpoint of treatment failure is measured at 24 months after the end of treatment. It is reassuring that to date there has been only one further relapse among the 47 who have reached this time point in the trial.

For both individuals with TB and national TB programs, a shorter duration of treatment that is effective is hugely beneficial. Visits to health care facilities place a financial and time burden on patients. In addition income loss often constitutes the largest financial risk for patients. For the TB program, a shorter duration of treatment translates into a lower number of patients in care at any one time with the potential to reduce loss to follow up.

There was a high rate of adverse events related to linezolid in the trial. Eighty percent of the participants reported peripheral neuropathy and almost half had evidence of haematological toxicity. While patients taking this regimen clearly need careful monitoring, these toxicities were manageable in this setting. All eight patients who had the regimen interrupted for hepatic adverse events, resumed and completed the full 26 weeks of treatment.

Nix-TB is the first study to treat XDR-TB and complicated MDR-TB with a regimen consisting of three oral agents for just 26 weeks. Despite historically being a hard to treat patient group, treatment success was 90%, which is higher than even those of the standard of care (HRZE) therapy in modern drug sensitive (DS) TB trials.[25-27]
