# Supplementary material for: Bedaquiline, pretomanid and linezolid for treatment of extensively drug resistant, intolerant or non-responsive multidrug resistant pulmonary tuberculosis
Source: N Engl J Med. Author manuscript; Available in PMC 2020 Mar 5. (PMC6955640; doi:10.1056/NEJMoa1901814)

Contents

Nix-TB Trial Team – additional names 2

Table S1 Anti-Retroviral Medications Taken by Patients in Nix-TB Trial During Treatment with the Investigational Regimen 3

Table S2 Screen Failure Reason for Individual Patients 4

Table S3 Deaths during the study 5

Table S4 Adverse events by system organ class and preferred term 6

Table S5 Serious adverse events by system organ class and preferred term 25

Figure S1 Time to first reduction or interruption of linezolid 28

Nix-TB Trial Team – additional names

Dr Nokuphiwa Mvuna, Sizwe Tropical Disease Hospital, Sandringham, South Africa

Dr Caryn Upton, Task Applied Science and Stellenbosch University, Cape Town, South Africa

Dr Naadira Vanker, Task Applied Science and Stellenbosch University, Cape Town, South Africa

Dr Lize Greyling, Task Applied Science and Stellenbosch University, Cape Town, South Africa

Ms Michelle Eriksson Task Applied Science and Stellenbosch University, Cape Town, South Africa

Stella Fabiane, MRC Clinical Trials Unit at UCL, London, UK

Julio Ortiz Canseco, UCL Centre for Clinical Microbiology, University College London, UK

Priya Solanki, UCL Centre for Clinical Microbiology, University College London, UK

Table S1 Anti-Retroviral Medications Taken by Patients in Nix-TB Trial During Treatment with the Investigational Regimen

Medication	Patients
Lopinavir/ritonavir	22
Nevirapine	17
Lamivudine	16
Emtricitabine/tenofovir	14
Tenofovir	9
Abacavir	8
Abacavir/lamivudine	8
Emtricitabine	4
Lopinavir/ritonavir	3
Stavudine	2
Abacavir/lamivudine	1
Atazanavir/ritonavir	1
Efavirenz	1
Lamivudine/zidovudine	1
Lamivudine/tenofovir	1

Table S2 Screen Failure Reason for Individual Patients

Patient	Reason for screen failure
1	CD4 < 50 (EXC 5)
2	RECEIVED TB TREATMENT WITHIN 3 DAYS PRIOR TO STUDY TREATMENT (EXC 15)
3	SPONSOR DECISION PATIENT RECEIVED > 2 WEEKS OF LZD AND BDQ.
4	QTCF > 500 (EXC 8)
5	RECEIVED TB TREATMENT WITHIN 3 DAYS PRIOR TO STUDY TREATMENT (EXC 15)
6	SIGNIFICANT PERIPHERAL NEUROPATHY (EXC 10)
7	XDR- NO DOCUMENTED TB TEST RESULTS CONFIRMING (INC 6)
8	ASPARTATE AMINOTRANSFERASE (AST) GREATER THAN OR EQUAL TO 5 TIMES ULN (EXC 16)
9	RECEIVED TB TREATMENT WITHIN 3 DAYS PRIOR TO STUDY TREATMENT (EXC 15)
10	EXCLUDED BY OPHTHALMOLOGY ON AREDS2 LENS GRADING
11	MEDICAL MONITOR ADVICED TO EXCLUDE PATIENT ON BASIS OF RAISED PRE-ENTRY LIPASE + AMYLASE, PANCREATITIS
12	POTASSIUM 3.0, ALT GRADE 3, AST GRADE3 AND ALBUMIN 3
13	SUBJECT COMPLETED MORE THAN 14 DAYS OF LINEZOLID
14	SIGNIFICANT PERIPHERAL NEUROPATHY (EXC 10)
15	PATIENT HAS DRUG SENSITIVE TB AND NO MDR-TB OR XDR TB
16	RECEIVED FLUCANAZOLE <30/7
17	UNABLE TO ENSURE 2 WEEK INTERVAL BETWEEN COMMENCING STUDY IMP AND START OF COMPATIBLE ART (EXC 5)
18	CD4 < 50 (EXC 5)
19	SERUM POTASSIUM LESS THAN LOWER LIMIT OF NORMAL (EXC 16)
20	DUE TO SCREENING WINDOW BEING EXCEEDED
21	HEMOGLOBIN LESS THAN 8.0 G/DL (EXC 16)
22	CLINICALLY SIGNIFICANT DISEASE OR ABNORMALITY (EXC 1)
23	RECEIVED TB TREATMENT WITHIN 3 DAYS PRIOR TO STUDY TREATMENT (EXC 15)
24	UNWILLING TO ATTEND VISITS/UNDERGO ASSESSMENTS (INC 3)
25	USE OF STRONG CYP450 ENZYME INDUCER WITHIN 30 DAYS PRIOR TO ENTRY
26	MDR-TB- NO DOCUMENTED NON-RESPONSE OR INTOLERANCE (INC 6)
27	SUBJECTS WITH OTHER CARDIAC ABNORMALITIES THAT MAY PLACE THEM AT RISK OF ARRHYTHMIA'S.
28	UNWILLING TO ATTEND VISITS/UNDERGO ASSESSMENTS (INC 3)
29	CLINICALLY SIGNIFICANT DISEASE OR ABNORMALITY (EXC 1)
30	CLINICALLY SIGNIFICANT DISEASE OR ABNORMALITY (EXC 1)
31	EXCLUSION 19
32	9 DAYS SCREENING ELLAPSED
33	REPEAT POTASSIUM WAS 3.0MMOL/L
34	LIVER TOXICITY GRADE 4 [RAISED ALANINE AMINOTRANSFERASE (ALT) AND ASPARTATE AMINOTRANSFERASE (AST)]

Table S3 Deaths during the study

Patient	Day of Death	Fatal SAEs (Preferred Term)	HIV Status
**1**	Day 35	Pulmonary tuberculosis<br>Disseminated tuberculosis	Positive
**2**	Day 51	Upper gastrointestinal haemorrhage	Negative
**3**	Day 55	Pulmonary tuberculosis	Positive
**4**	Day 53	Pancreatitis haemorrhagic<br>Multiple organ dysfunction syndrome	Positive
**5**	Day 93	Sepsis<br>Pneumonia	Negative
**6**	Day 76	Septic shock<br>Pneumonia	Negative
**7**	Day 369 (185 days after EOT)	Natural causes	Positive
**8***	Day 486 (303 days after EOT)	Thrombotic thrombocytopenic purpura<br>Sepsis<br>Dry gangrene<br>Peripheral vascular disorder<br>Infected skin ulcer	Positive

Table S4 Adverse events by system organ class and preferred term

Table S4
Incidence of TEAEs by Severity and Relationship
Population: Safety

MedDRA System Organ Class / Preferred Term	Grade 1		Grade 2		Grade 3		Grade 4		Any		Total
	Related	Not Related	Related	Not Related	Related	Not Related	Related	Not Related	Related	Not Related	R+NR
NERVOUS SYSTEM DISORDERS	55(50.5)	13(11.9)	45(41.3)	5(4.6)	24(22.0)	4(3.7)	1(0.9)	0	91(83.5)	20(18.3)	92(84.4)
#PERIPHERAL SENSORY NEUROPATHY	29(26.6)	0	38(34.9)	1(0.9)	20(18.3)	0	0	0	76(69.7)	1(0.9)	76(69.7)
HEADACHE	20(18.3)	7(6.4)	2(1.8)	1(0.9)	0	1(0.9)	0	0	22(20.2)	9(8.3)	30(27.5)
#NEUROPATHY PERIPHERAL	4(3.7)	0	7(6.4)	0	3(2.8)	0	0	0	10(9.2)	0	10(9.2)
DIZZINESS	3(2.8)	2(1.8)	0	0	0	0	0	0	3(2.8)	2(1.8)	5(4.6)
DYSGEUSIA	5(4.6)	0	0	0	0	0	0	0	5(4.6)	0	5(4.6)
#PARAESTHESIA	5(4.6)	0	0	0	0	0	0	0	5(4.6)	0	5(4.6)
#HYPOAESTHESIA	3(2.8)	0	0	0	0	0	0	0	3(2.8)	0	3(2.8)
#PERIPHERAL SENSORIMOTOR NEUROPATHY	2(1.8)	0	0	0	1(0.9)	0	0	0	3(2.8)	0	3(2.8)
#BURNING SENSATION	2(1.8)	0	0	0	0	0	0	0	2(1.8)	0	2(1.8)
#PERIPHERAL MOTOR NEUROPATHY	2(1.8)	0	0	0	0	0	0	0	2(1.8)	0	2(1.8)
CHRONIC INFLAMMATORY DEMYELINATING POLYRADICULONEUROPATHY	0	0	0	0	0	1(0.9)	0	0	0	1(0.9)	1(0.9)
DIZZINESS POSTURAL	0	0	0	1(0.9)	0	0	0	0	0	1(0.9)	1(0.9)
DYSTONIA	0	1(0.9)	0	0	0	0	0	0	0	1(0.9)	1(0.9)
#GENERALISED TONIC-CLONIC SEIZURE	0	0	0	0	0	1(0.9)	0	0	0	1(0.9)	1(0.9)
#HYPOREFLEXIA	1(0.9)	0	0	0	0	0	0	0	1(0.9)	0	1(0.9)
MIGRAINE	0	0	0	1(0.9)	0	0	0	0	0	1(0.9)	1(0.9)
#OPTIC NEURITIS	0	0	0	0	0	0	1(0.9)	0	1(0.9)	0	1(0.9)

MedDRA: Medical Dictionary for Regulatory Activities. NR: Not related. R: Related. n = Number of subjects with at least one TEAE in each category (subjects with multiple AEs in each category are counted only once in each category). N = Total number of subjects in the relevant analysis population. % = Percentage of subjects with at least one TEAE in each category relative to the total number of subjects in the relevant analysis population. Treatment-emergent adverse events (TEAEs): Defined as AEs which started or worsened on or after the first study drug administration up to and including 14 days after the last study drug administration. Grade I,II,III, IV TEAEs: Defined as TEAEs for which the severity (DMID grade) is indicated as Grade 1 (mild), Grade 2 (moderate), Grade 3 (severe) and Grade 4 (potentially life-threatening) respectively. #: Indicates TEAEs of special interest. TEAEs of special interest: Identified by pre-specified SMQ codes as confirmed by TB Alliance . Adverse events: Coded using MedDRA Version 20.0.

Program: PRODUCTION\TABLES\PEL\T14_3_1_11_PEL.SAS (18JUN2019 12:32) File: T14_3_1_11_PEL.rtf

Table S4
Incidence of TEAEs by Severity and Relationship
Population: Safety

MedDRA System Organ Class<br>Preferred Term	Grade 1		Grade 2		Grade 3		Grade 4		Any		Total
	Related	Not Related	Related	Not Related	Related	Not Related	Related	Not Related	Related	Not Related	R+NR
NERVOUS SYSTEM DISORDERS											
#SEIZURE	0	0	0	0	0	1(0.9)	0	0	0	1(0.9)	1(0.9)
SINUS HEADACHE	0	1(0.9)	0	0	0	0	0	0	0	1(0.9)	1(0.9)
#SYNCOPE	0	0	0	1(0.9)	0	1(0.9)	0	0	0	1(0.9)	1(0.9)
TENSION HEADACHE	0	1(0.9)	0	0	0	0	0	0	0	1(0.9)	1(0.9)
TREMOR	0	1(0.9)	0	0	0	0	0	0	0	1(0.9)	1(0.9)
GASTROINTESTINAL DISORDERS	54(49.5)	37(33.9)	17(15.6)	8(7.3)	3(2.8)	1(0.9)	2(1.8)	0	60(55.0)	40(36.7)	73(67.0)
NAUSEA	35(32.1)	2(1.8)	8(7.3)	1(0.9)	0	0	0	0	39(35.8)	3(2.8)	40(36.7)
VOMITING	22(20.2)	9(8.3)	10(9.2)	3(2.8)	0	0	0	0	31(28.4)	10(9.2)	37(33.9)
DYSPEPSIA	18(16.5)	8(7.3)	1(0.9)	1(0.9)	0	0	0	0	19(17.4)	9(8.3)	26(23.9)
ABDOMINAL PAIN	4(3.7)	3(2.8)	3(2.8)	1(0.9)	0	0	0	0	7(6.4)	4(3.7)	11(10.1)
DIARRHOEA	6(5.5)	6(5.5)	0	0	0	0	0	0	6(5.5)	6(5.5)	11(10.1)
CONSTIPATION	4(3.7)	4(3.7)	0	1(0.9)	0	0	0	0	4(3.7)	5(4.6)	9(8.3)
GASTRITIS	5(4.6)	2(1.8)	4(3.7)	0	0	0	0	0	8(7.3)	2(1.8)	9(8.3)
ABDOMINAL PAIN UPPER	5(4.6)	0	2(1.8)	0	2(1.8)	0	0	0	7(6.4)	0	7(6.4)
ABDOMINAL PAIN LOWER	1(0.9)	2(1.8)	0	0	0	0	0	0	1(0.9)	2(1.8)	3(2.8)
ABDOMINAL TENDERNESS	3(2.8)	0	0	0	0	0	0	0	3(2.8)	0	3(2.8)
GASTROOESOPHAGEAL REFLUX DISEASE	2(1.8)	1(0.9)	0	1(0.9)	0	0	0	0	2(1.8)	1(0.9)	3(2.8)
HAEMATEMESIS	0	1(0.9)	1(0.9)	0	0	1(0.9)	0	0	1(0.9)	2(1.8)	3(2.8)

MedDRA: Medical Dictionary for Regulatory Activities. NR: Not related. R: Related. n = Number of subjects with at least one TEAE in each category (subjects with multiple AEs in each category are counted only once in each category). N = Total number of subjects in the relevant analysis population. % = Percentage of subjects with at least one TEAE in each category relative to the total number of subjects in the relevant analysis population. Treatment-emergent adverse events (TEAEs): Defined as AEs which started or worsened on or after the first study drug administration up to and including 14 days after the last study drug administration. Grade I,II,III, IV TEAEs: Defined as TEAEs for which the severity (DMID grade) is indicated as Grade 1 (mild), Grade 2 (moderate), Grade 3 (severe) and Grade 4 (potentially life-threatening) respectively. #: Indicates TEAEs of special interest. TEAEs of special interest: Identified by pre-specified SMQ codes as confirmed by TB Alliance . Adverse events: Coded using MedDRA Version 20.0.

Program: PRODUCTION\TABLES\PEL\T14_3_1_11_PEL.SAS (18JUN2019 12:32) File: T14_3_1_11_PEL.rtf

Table S4
Incidence of TEAEs by Severity and Relationship
Population: Safety

MedDRA System Organ Class / Preferred Term	Grade 1 Related	Grade 1 Not Related	Grade 2 Related	Grade 2 Not Related	Grade 3 Related	Grade 3 Not Related	Grade 4 Related	Grade 4 Not Related	Any Related	Any Not Related	Total R+NR
GASTROINTESTINAL DISORDERS											
HAEMORRHOIDS	0	3(2.8)	0	0	0	0	0	0	0	3(2.8)	3(2.8)
GLOSSODYNIA	1(0.9)	1(0.9)	0	0	0	0	0	0	1(0.9)	1(0.9)	2(1.8)
HAEMORRHOIDAL HAEMORRHAGE	0	2(1.8)	0	0	0	0	0	0	0	2(1.8)	2(1.8)
#PANCREATITIS	0	0	1(0.9)	0	1(0.9)	0	0	0	2(1.8)	0	2(1.8)
TOOTHACHE	0	2(1.8)	0	0	0	0	0	0	0	2(1.8)	2(1.8)
UPPER GASTROINTESTINAL HAEMORRHAGE	0	0	0	0	0	0	2(1.8)	0	2(1.8)	0	2(1.8)
ABDOMINAL DISCOMFORT	0	0	1(0.9)	0	0	0	0	0	1(0.9)	0	1(0.9)
ABDOMINAL DISTENSION	1(0.9)	0	0	0	0	0	0	0	1(0.9)	0	1(0.9)
ATROPHIC GLOSSITIS	0	1(0.9)	0	0	0	0	0	0	0	1(0.9)	1(0.9)
CHRONIC GASTRITIS	1(0.9)	0	0	0	0	0	0	0	1(0.9)	0	1(0.9)
DENTAL CARIES	0	0	0	1(0.9)	0	0	0	0	0	1(0.9)	1(0.9)
DYSPHAGIA	0	1(0.9)	0	0	0	0	0	0	0	1(0.9)	1(0.9)
FAECALOMA	0	0	1(0.9)	0	0	0	0	0	1(0.9)	0	1(0.9)
GASTRIC POLYPS	0	1(0.9)	0	0	0	0	0	0	0	1(0.9)	1(0.9)
HAEMATOCHEZIA	0	1(0.9)	0	0	0	0	0	0	0	1(0.9)	1(0.9)
HIATUS HERNIA	0	1(0.9)	0	0	0	0	0	0	0	1(0.9)	1(0.9)
HYPERTROPHY OF TONGUE PAPILLAE	1(0.9)	0	0	0	0	0	0	0	1(0.9)	0	1(0.9)
MELAENA	0	1(0.9)	0	0	0	0	0	0	0	1(0.9)	1(0.9)

MedDRA: Medical Dictionary for Regulatory Activities. NR: Not related. R: Related. n = Number of subjects with at least one TEAE in each category (subjects with multiple AEs in each category are counted only once in each category). N = Total number of subjects in the relevant analysis population. % = Percentage of subjects with at least one TEAE in each category relative to the total number of subjects in the relevant analysis population. Treatment-emergent adverse events (TEAEs): Defined as AEs which started or worsened on or after the first study drug administration up to and including 14 days after the last study drug administration. Grade I,II,III, IV TEAEs: Defined as TEAEs for which the severity (DMID grade) is indicated as Grade 1 (mild), Grade 2 (moderate), Grade 3 (severe) and Grade 4 (potentially life-threatening) respectively. #: Indicates TEAEs of special interest. TEAEs of special interest: Identified by pre-specified SMQ codes as confirmed by TB Alliance . Adverse events: Coded using MedDRA Version 20.0.

Program: PRODUCTION\TABLES\PEL\T14 3 1 11 PEL.SAS (18JUN2019 12:32) File: T14 3 1 11 PEL.rtf

Table S4
Incidence of TEAEs by Severity and Relationship
Population: Safety

MedDRA System Organ Class<br>Preferred Term	Grade 1<br>Related	 Not Related	Grade 2<br>Related	 Not Related	Grade 3<br>Related	 Not Related	Grade 4<br>Related	 Not Related	Any<br>Related	 Not Related	Total<br>R+NR
GASTROINTESTINAL DISORDERS											
MOUTH ULCERATION	0	1(0.9)	0	0	0	0	0	0	0	1(0.9)	1(0.9)
ORAL DISORDER	0	1(0.9)	0	0	0	0	0	0	0	1(0.9)	1(0.9)
#PANCREATITIS HAEMORRHAGIC	0	0	0	0	0	0	1(0.9)	0	1(0.9)	0	1(0.9)
PROCTALGIA	0	1(0.9)	0	0	0	0	0	0	0	1(0.9)	1(0.9)
STOMATITIS	0	1(0.9)	0	0	0	0	0	0	0	1(0.9)	1(0.9)
TONGUE ULCERATION	0	1(0.9)	0	0	0	0	0	0	0	1(0.9)	1(0.9)
TOOTH DISCOLOURATION	0	1(0.9)	0	0	0	0	0	0	0	1(0.9)	1(0.9)
TOOTH LOSS	0	1(0.9)	0	0	0	0	0	0	0	1(0.9)	1(0.9)
SKIN AND SUBCUTANEOUS TISSUE DISORDERS	45(41.3)	31(28.4)	3(2.8)	4(3.7)	0	0	0	0	47(43.1)	34(31.2)	69(63.3)
DERMATITIS ACNEIFORM	16(14.7)	8(7.3)	1(0.9)	1(0.9)	0	0	0	0	17(15.6)	9(8.3)	26(23.9)
RASH	11(10.1)	8(7.3)	0	2(1.8)	0	0	0	0	11(10.1)	9(8.3)	18(16.5)
ACNE	16(14.7)	1(0.9)	0	0	0	0	0	0	16(14.7)	1(0.9)	17(15.6)
PRURITUS	11(10.1)	7(6.4)	0	0	0	0	0	0	11(10.1)	7(6.4)	16(14.7)
DRY SKIN	5(4.6)	4(3.7)	0	0	0	0	0	0	5(4.6)	4(3.7)	8(7.3)
RASH PRURITIC	0	3(2.8)	1(0.9)	0	0	0	0	0	1(0.9)	3(2.8)	4(3.7)
RASH PAPULAR	1(0.9)	2(1.8)	0	0	0	0	0	0	1(0.9)	2(1.8)	3(2.8)
SKIN HYPERPIGMENTATION	1(0.9)	1(0.9)	0	1(0.9)	0	0	0	0	1(0.9)	2(1.8)	3(2.8)
ALOPECIA	2(1.8)	0	0	0	0	0	0	0	2(1.8)	0	2(1.8)

MedDRA: Medical Dictionary for Regulatory Activities. NR: Not related. R: Related. n = Number of subjects with at least one TEAE in each category (subjects with multiple AEs in each category are counted only once in each category). N = Total number of subjects in the relevant analysis population. % = Percentage of subjects with at least one TEAE in each category relative to the total number of subjects in the relevant analysis population. Treatment-emergent adverse events (TEAEs): Defined as AEs which started or worsened on or after the first study drug administration up to and including 14 days after the last study drug administration. Grade I,II,III, IV TEAEs: Defined as TEAEs for which the severity (DMID grade) is indicated as Grade 1 (mild), Grade 2 (moderate), Grade 3 (severe) and Grade 4 (potentially life-threatening) respectively. #: Indicates TEAEs of special interest. TEAEs of special interest: Identified by pre-specified SMQ codes as confirmed by TB Alliance . Adverse events: Coded using MedDRA Version 20.0.

Program: PRODUCTION\TABLES\PEL\T14 3 1 11 PEL.SAS (18JUN2019 12:32) File: T14 3 1 11 PEL.rtf

Table S4
Incidence of TEAEs by Severity and Relationship
Population: Safety

MedDRA System Organ Class<br>Preferred Term	Grade 1<br>Related	Grade 1<br>Not Related	Grade 2<br>Related	Grade 2<br>Not Related	Grade 3<br>Related	Grade 3<br>Not Related	Grade 4<br>Related	Grade 4<br>Not Related	Any<br>Related	Any<br>Not Related	Total<br>R+NR
SKIN AND SUBCUTANEOUS TISSUE DISORDERS											
PRURITUS GENERALISED	0	1(0.9)	0	1(0.9)	0	0	0	0	0	2(1.8)	2(1.8)
RASH MACULO-PAPULAR	1(0.9)	0	1(0.9)	0	0	0	0	0	2(1.8)	0	2(1.8)
DERMATITIS ALLERGIC	1(0.9)	0	0	0	0	0	0	0	1(0.9)	0	1(0.9)
DERMATITIS ATOPIC	0	1(0.9)	0	0	0	0	0	0	0	1(0.9)	1(0.9)
DERMATITIS CONTACT	0	1(0.9)	0	0	0	0	0	0	0	1(0.9)	1(0.9)
NAIL DYSTROPHY	0	1(0.9)	0	0	0	0	0	0	0	1(0.9)	1(0.9)
PENILE ULCERATION	0	1(0.9)	0	0	0	0	0	0	0	1(0.9)	1(0.9)
RASH ERYTHEMATOUS	1(0.9)	0	0	0	0	0	0	0	1(0.9)	0	1(0.9)
RASH MACULAR	0	1(0.9)	0	0	0	0	0	0	0	1(0.9)	1(0.9)
RASH VESICULAR	0	1(0.9)	0	0	0	0	0	0	0	1(0.9)	1(0.9)
SKIN DISCOLOURATION	0	1(0.9)	0	0	0	0	0	0	0	1(0.9)	1(0.9)
SKIN MASS	0	1(0.9)	0	0	0	0	0	0	0	1(0.9)	1(0.9)
SKIN ULCER	0	1(0.9)	0	0	0	0	0	0	0	1(0.9)	1(0.9)
URTICARIA	0	1(0.9)	0	0	0	0	0	0	0	1(0.9)	1(0.9)
INFECTIONS AND INFESTATIONS	5(4.6)	57(52.3)	0	18(16.5)	0	4(3.7)	0	6(5.5)	5(4.6)	63(57.8)	64(58.7)
UPPER RESPIRATORY TRACT INFECTION	0	19(17.4)	0	2(1.8)	0	1(0.9)	0	0	0	21(19.3)	21(19.3)
LOWER RESPIRATORY TRACT INFECTION	0	7(6.4)	0	1(0.9)	0	0	0	0	0	7(6.4)	7(6.4)
URINARY TRACT INFECTION	0	5(4.6)	0	2(1.8)	0	0	0	0	0	7(6.4)	7(6.4)

MedDRA: Medical Dictionary for Regulatory Activities. NR: Not related. R: Related. n = Number of subjects with at least one TEAE in each category (subjects with multiple AEs in each category are counted only once in each category). N = Total number of subjects in the relevant analysis population. % = Percentage of subjects with at least one TEAE in each category relative to the total number of subjects in the relevant analysis population. Treatment-emergent adverse events (TEAEs): Defined as AEs which started or worsened on or after the first study drug administration up to and including 14 days after the last study drug administration. Grade I,II,III, IV TEAEs: Defined as TEAEs for which the severity (DMID grade) is indicated as Grade 1 (mild), Grade 2 (moderate), Grade 3 (severe) and Grade 4 (potentially life-threatening) respectively. #: Indicates TEAEs of special interest. TEAEs of special interest: Identified by pre-specified SMQ codes as confirmed by TB Alliance . Adverse events: Coded using MedDRA Version 20.0.

Program: PRODUCTION\TABLES\PEL\T14_3_1_11_PEL.SAS (18JUN2019 12:32) File: T14_3_1_11_PEL.rtf

Table S4
Incidence of TEAEs by Severity and Relationship
Population: Safety

MedDRA System Organ Class Preferred Term	Grade 1 Related	Grade 1 Not Related	Grade 2 Related	Grade 2 Not Related	Grade 3 Related	Grade 3 Not Related	Grade 4 Related	Grade 4 Not Related	Any Related	Any Not Related	Total R+NR
INFECTIONS AND INFESTATIONS											
INFLUENZA	0	6(5.5)	0	0	0	0	0	0	0	6(5.5)	6(5.5)
ANGULAR CHEILITIS	1(0.9)	4(3.7)	0	0	0	0	0	0	1(0.9)	4(3.7)	5(4.6)
ORAL CANDIDIASIS	2(1.8)	2(1.8)	0	0	0	1(0.9)	0	0	2(1.8)	3(2.8)	5(4.6)
TINEA INFECTION	1(0.9)	3(2.8)	0	1(0.9)	0	0	0	0	1(0.9)	4(3.7)	5(4.6)
CONJUNCTIVITIS	1(0.9)	3(2.8)	0	0	0	0	0	0	1(0.9)	3(2.8)	4(3.7)
PNEUMONIA	0	1(0.9)	0	1(0.9)	0	2(1.8)	0	2(1.8)	0	4(3.7)	4(3.7)
FUNGAL SKIN INFECTION	0	3(2.8)	0	0	0	0	0	0	0	3(2.8)	3(2.8)
PULMONARY TUBERCULOSIS	0	0	0	1(0.9)	0	0	0	2(1.8)	0	3(2.8)	3(2.8)
TINEA CRURIS	0	2(1.8)	0	1(0.9)	0	0	0	0	0	3(2.8)	3(2.8)
TINEA PEDIS	0	2(1.8)	0	1(0.9)	0	0	0	0	0	3(2.8)	3(2.8)
TINEA VERSICOLOUR	0	2(1.8)	0	1(0.9)	0	0	0	0	0	3(2.8)	3(2.8)
VIRAL UPPER RESPIRATORY TRACT INFECTION	0	3(2.8)	0	0	0	0	0	0	0	3(2.8)	3(2.8)
ABSCESS LIMB	0	0	0	2(1.8)	0	0	0	0	0	2(1.8)	2(1.8)
ACARODERMATITIS	0	2(1.8)	0	0	0	0	0	0	0	2(1.8)	2(1.8)
GASTROENTERITIS	0	2(1.8)	0	0	0	0	0	0	0	2(1.8)	2(1.8)
GASTROENTERITIS VIRAL	0	2(1.8)	0	0	0	0	0	0	0	2(1.8)	2(1.8)
SEPSIS	0	0	0	0	0	0	0	2(1.8)	0	2(1.8)	2(1.8)
TINEA CAPITIS	0	2(1.8)	0	0	0	0	0	0	0	2(1.8)	2(1.8)

MedDRA: Medical Dictionary for Regulatory Activities. NR: Not related. R: Related. n = Number of subjects with at least one TEAE in each category (subjects with multiple AEs in each category are counted only once in each category). N = Total number of subjects in the relevant analysis population. % = Percentage of subjects with at least one TEAE in each category relative to the total number of subjects in the relevant analysis population. Treatment-emergent adverse events (TEAEs): Defined as AEs which started or worsened on or after the first study drug administration up to and including 14 days after the last study drug administration. Grade I,II,III, IV TEAEs: Defined as TEAEs for which the severity (DMID grade) is indicated as Grade 1 (mild), Grade 2 (moderate), Grade 3 (severe) and Grade 4 (potentially life-threatening) respectively. #: Indicates TEAEs of special interest. TEAEs of special interest: Identified by pre-specified SMQ codes as confirmed by TB Alliance . Adverse events: Coded using MedDRA Version 20.0.

Program: PRODUCTION\TABLES\PEL\T14 3 1 11 PEL.SAS (18JUN2019 12:32) File: T14 3 1 11 PEL.rtf

Table S4
Incidence of TEAEs by Severity and Relationship
Population: Safety

MedDRA System Organ Class<br>Preferred Term	Grade 1<br>Related	Grade 1<br>Not Related	Grade 2<br>Related	Grade 2<br>Not Related	Grade 3<br>Related	Grade 3<br>Not Related	Grade 4<br>Related	Grade 4<br>Not Related	Any<br>Related	Any<br>Not Related	Total<br>R+NR
INFECTIONS AND INFESTATIONS											
TONSILLITIS	0	1(0.9)	0	1(0.9)	0	0	0	0	0	2(1.8)	2(1.8)
ABSCESS	0	0	0	1(0.9)	0	0	0	0	0	1(0.9)	1(0.9)
BACTERIAL VAGINOSIS	0	0	0	1(0.9)	0	0	0	0	0	1(0.9)	1(0.9)
BALANITIS CANDIDA	0	1(0.9)	0	0	0	0	0	0	0	1(0.9)	1(0.9)
BRONCHITIS	0	1(0.9)	0	0	0	0	0	0	0	1(0.9)	1(0.9)
CELLULITIS	0	0	0	1(0.9)	0	0	0	0	0	1(0.9)	1(0.9)
CONJUNCTIVITIS BACTERIAL	0	1(0.9)	0	0	0	0	0	0	0	1(0.9)	1(0.9)
DEVICE RELATED SEPSIS	0	1(0.9)	0	0	0	0	0	0	0	1(0.9)	1(0.9)
DISSEMINATED TUBERCULOSIS	0	0	0	0	0	0	0	1(0.9)	0	1(0.9)	1(0.9)
DYSENTERY	0	1(0.9)	0	0	0	0	0	0	0	1(0.9)	1(0.9)
EYE INFECTION	0	1(0.9)	0	0	0	0	0	0	0	1(0.9)	1(0.9)
FUNGAL INFECTION	1(0.9)	0	0	0	0	0	0	0	1(0.9)	0	1(0.9)
GENITAL CANDIDIASIS	0	1(0.9)	0	0	0	0	0	0	0	1(0.9)	1(0.9)
GENITAL ULCER SYNDROME	0	1(0.9)	0	0	0	0	0	0	0	1(0.9)	1(0.9)
HERPES SIMPLEX	0	1(0.9)	0	0	0	0	0	0	0	1(0.9)	1(0.9)
LYMPH NODE TUBERCULOSIS	0	0	0	1(0.9)	0	0	0	0	0	1(0.9)	1(0.9)
ORAL FUNGAL INFECTION	1(0.9)	0	0	0	0	0	0	0	1(0.9)	0	1(0.9)
ORAL HERPES	0	1(0.9)	0	0	0	0	0	0	0	1(0.9)	1(0.9)

MedDRA: Medical Dictionary for Regulatory Activities. NR: Not related. R: Related. n = Number of subjects with at least one TEAE in each category (subjects with multiple AEs in each category are counted only once in each category). N = Total number of subjects in the relevant analysis population. % = Percentage of subjects with at least one TEAE in each category relative to the total number of subjects in the relevant analysis population. Treatment-emergent adverse events (TEAEs): Defined as AEs which started or worsened on or after the first study drug administration up to and including 14 days after the last study drug administration. Grade I,II,III, IV TEAEs: Defined as TEAEs for which the severity (DMID grade) is indicated as Grade 1 (mild), Grade 2 (moderate), Grade 3 (severe) and Grade 4 (potentially life-threatening) respectively. #: Indicates TEAEs of special interest. TEAEs of special interest: Identified by pre-specified SMQ codes as confirmed by TB Alliance . Adverse events: Coded using MedDRA Version 20.0.

Program: PRODUCTION\TABLES\PEL\T14 3 1 11 PEL.SAS (18JUN2019 12:32) File: T14 3 1 11 PEL.rtf

Table S4
Incidence of TEAEs by Severity and Relationship
Population: Safety

MedDRA System Organ Class<br>Preferred Term	Grade 1<br>Related	Grade 1<br>Not Related	Grade 2<br>Related	Grade 2<br>Not Related	Grade 3<br>Related	Grade 3<br>Not Related	Grade 4<br>Related	Grade 4<br>Not Related	Any<br>Related	Any<br>Not Related	Total<br>R+NR
INFECTIONS AND INFESTATIONS											
OTITIS EXTERNA	0	1(0.9)	0	0	0	0	0	0	0	1(0.9)	1(0.9)
OTITIS MEDIA	0	0	0	1(0.9)	0	0	0	0	0	1(0.9)	1(0.9)
OTITIS MEDIA ACUTE	0	0	0	1(0.9)	0	0	0	0	0	1(0.9)	1(0.9)
OTITIS MEDIA CHRONIC	0	1(0.9)	0	0	0	0	0	0	0	1(0.9)	1(0.9)
PHARYNGITIS	0	1(0.9)	0	0	0	0	0	0	0	1(0.9)	1(0.9)
SEPTIC SHOCK	0	0	0	0	0	0	0	1(0.9)	0	1(0.9)	1(0.9)
SKIN BACTERIAL INFECTION	0	0	0	1(0.9)	0	0	0	0	0	1(0.9)	1(0.9)
SKIN CANDIDA	0	1(0.9)	0	0	0	0	0	0	0	1(0.9)	1(0.9)
TUBERCULOMA OF CENTRAL NERVOUS SYSTEM	0	0	0	0	0	0	0	1(0.9)	0	1(0.9)	1(0.9)
TUBERCULOSIS	0	0	0	0	0	1(0.9)	0	0	0	1(0.9)	1(0.9)
VIRAL INFECTION	0	1(0.9)	0	0	0	0	0	0	0	1(0.9)	1(0.9)
VULVOVAGINAL CANDIDIASIS	0	1(0.9)	0	0	0	0	0	0	0	1(0.9)	1(0.9)
BLOOD AND LYMPHATIC SYSTEM DISORDERS	20(18.3)	0	26(23.9)	3(2.8)	9(8.3)	2(1.8)	3(2.8)	0	49(45.0)	5(4.6)	53(48.6)
#ANAEMIA	15(13.8)	0	19(17.4)	1(0.9)	4(3.7)	1(0.9)	2(1.8)	0	38(34.9)	2(1.8)	40(36.7)
#NEUTROPENIA	1(0.9)	0	4(3.7)	1(0.9)	3(2.8)	0	1(0.9)	0	8(7.3)	1(0.9)	9(8.3)
#THROMBOCYTOPENIA	4(3.7)	0	1(0.9)	0	1(0.9)	0	0	0	6(5.5)	0	6(5.5)
#BONE MARROW FAILURE	0	0	1(0.9)	0	2(1.8)	0	0	0	3(2.8)	0	3(2.8)
#LEUKOPENIA	1(0.9)	0	0	0	1(0.9)	0	0	0	2(1.8)	0	2(1.8)

MedDRA: Medical Dictionary for Regulatory Activities. NR: Not related. R: Related. n = Number of subjects with at least one TEAE in each category (subjects with multiple AEs in each category are counted only once in each category). N = Total number of subjects in the relevant analysis population. % = Percentage of subjects with at least one TEAE in each category relative to the total number of subjects in the relevant analysis population. Treatment-emergent adverse events (TEAEs): Defined as AEs which started or worsened on or after the first study drug administration up to and including 14 days after the last study drug administration. Grade I,II,III, IV TEAEs: Defined as TEAEs for which the severity (DMID grade) is indicated as Grade 1 (mild), Grade 2 (moderate), Grade 3 (severe) and Grade 4 (potentially life-threatening) respectively. #: Indicates TEAEs of special interest. TEAEs of special interest: Identified by pre-specified SMQ codes as confirmed by TB Alliance . Adverse events: Coded using MedDRA Version 20.0.

Program: PRODUCTION\TABLES\PEL\T14 3 1 11 PEL.SAS (18JUN2019 12:32) File: T14 3 1 11 PEL.rtf

Table S4
Incidence of TEAEs by Severity and Relationship
Population: Safety

MedDRA System Organ Class / Preferred Term	Grade 1 Related	Grade 1 Not Related	Grade 2 Related	Grade 2 Not Related	Grade 3 Related	Grade 3 Not Related	Grade 4 Related	Grade 4 Not Related	Any Related	Any Not Related	Total R+NR
BLOOD AND LYMPHATIC SYSTEM DISORDERS											
#BICYTOPENIA	0	0	1(0.9)	0	0	0	0	0	1(0.9)	0	1(0.9)
DISSEMINATED INTRAVASCULAR COAGULATION	0	0	0	1(0.9)	0	0	0	0	0	1(0.9)	1(0.9)
LEUKOCYTOSIS	0	0	0	0	0	1(0.9)	0	0	0	1(0.9)	1(0.9)
#LYMPHOPENIA	0	0	1(0.9)	0	0	0	0	0	1(0.9)	0	1(0.9)
#PANCYTOPENIA	1(0.9)	0	0	0	0	0	0	0	1(0.9)	0	1(0.9)
RESPIRATORY, THORACIC AND MEDIASTINAL DISORDERS	5(4.6)	50(45.9)	1(0.9)	8(7.3)	1(0.9)	1(0.9)	0	2(1.8)	7(6.4)	51(46.8)	53(48.6)
PLEURITIC PAIN	2(1.8)	18(16.5)	0	1(0.9)	1(0.9)	0	0	0	3(2.8)	19(17.4)	21(19.3)
HAEMOPTYSIS	1(0.9)	12(11.0)	1(0.9)	1(0.9)	0	0	0	1(0.9)	2(1.8)	12(11.0)	14(12.8)
COUGH	0	9(8.3)	0	0	0	0	0	0	0	9(8.3)	9(8.3)
CHRONIC OBSTRUCTIVE PULMONARY DISEASE	0	5(4.6)	0	3(2.8)	0	0	0	0	0	8(7.3)	8(7.3)
BRONCHOSPASM	0	5(4.6)	0	0	0	0	0	0	0	5(4.6)	5(4.6)
DYSPNOEA	0	1(0.9)	0	2(1.8)	0	1(0.9)	0	1(0.9)	0	5(4.6)	5(4.6)
PRODUCTIVE COUGH	1(0.9)	4(3.7)	0	0	0	0	0	0	1(0.9)	4(3.7)	5(4.6)
NASAL CONGESTION	0	4(3.7)	0	0	0	0	0	0	0	4(3.7)	4(3.7)
RHINORRHOEA	0	4(3.7)	0	0	0	0	0	0	0	4(3.7)	4(3.7)
EPISTAXIS	1(0.9)	2(1.8)	0	0	0	0	0	0	1(0.9)	2(1.8)	3(2.8)
PULMONARY PAIN	0	3(2.8)	0	0	0	0	0	0	0	3(2.8)	3(2.8)
ASTHMA	0	0	0	1(0.9)	0	1(0.9)	0	0	0	2(1.8)	2(1.8)

MedDRA: Medical Dictionary for Regulatory Activities. NR: Not related. R: Related. n = Number of subjects with at least one TEAE in each category (subjects with multiple AEs in each category are counted only once in each category). N = Total number of subjects in the relevant analysis population. % = Percentage of subjects with at least one TEAE in each category relative to the total number of subjects in the relevant analysis population. Treatment-emergent adverse events (TEAEs): Defined as AEs which started or worsened on or after the first study drug administration up to and including 14 days after the last study drug administration. Grade I,II,III, IV TEAEs: Defined as TEAEs for which the severity (DMID grade) is indicated as Grade 1 (mild), Grade 2 (moderate), Grade 3 (severe) and Grade 4 (potentially life-threatening) respectively. #: Indicates TEAEs of special interest. TEAEs of special interest: Identified by pre-specified SMQ codes as confirmed by TB Alliance . Adverse events: Coded using MedDRA Version 20.0.

Program: PRODUCTION\TABLES\PEL\T14 3 1 11 PEL.SAS (18JUN2019 12:32) File: T14 3 1 11 PEL.rtf

Table S4
Incidence of TEAEs by Severity and Relationship
Population: Safety

MedDRA System Organ Class / Preferred Term	Grade 1 Related	Grade 1 Not Related	Grade 2 Related	Grade 2 Not Related	Grade 3 Related	Grade 3 Not Related	Grade 4 Related	Grade 4 Not Related	Any Related	Any Not Related	Total R+NR
RESPIRATORY, THORACIC AND MEDIASTINAL DISORDERS											
OROPHARYNGEAL PAIN	0	2(1.8)	0	0	0	0	0	0	0	2(1.8)	2(1.8)
RHINITIS ALLERGIC	0	2(1.8)	0	0	0	0	0	0	0	2(1.8)	2(1.8)
PNEUMOTHORAX SPONTANEOUS	0	0	0	0	0	0	0	1(0.9)	0	1(0.9)	1(0.9)
RESPIRATORY DISTRESS	0	0	0	1(0.9)	0	0	0	0	0	1(0.9)	1(0.9)
INVESTIGATIONS	20(18.3)	8(7.3)	18(16.5)	6(5.5)	16(14.7)	7(6.4)	2(1.8)	2(1.8)	41(37.6)	20(18.3)	51(46.8)
#GAMMA-GLUTAMYLTRANSFERASE INCREASED	2(1.8)	0	9(8.3)	0	3(2.8)	3(2.8)	2(1.8)	1(0.9)	16(14.7)	4(3.7)	20(18.3)
#TRANSAMINASES INCREASED	2(1.8)	0	2(1.8)	0	5(4.6)	2(1.8)	0	1(0.9)	9(8.3)	3(2.8)	12(11.0)
#ALANINE AMINOTRANSFERASE INCREASED	2(1.8)	1(0.9)	5(4.6)	0	2(1.8)	0	0	0	9(8.3)	1(0.9)	10(9.2)
AMYLASE INCREASED	2(1.8)	0	1(0.9)	0	4(3.7)	2(1.8)	0	0	7(6.4)	2(1.8)	9(8.3)
#ASPARTATE AMINOTRANSFERASE INCREASED	2(1.8)	1(0.9)	3(2.8)	1(0.9)	2(1.8)	0	0	0	7(6.4)	2(1.8)	9(8.3)
#ELECTROCARDIOGRAM QT PROLONGED	6(5.5)	0	0	0	0	0	0	0	6(5.5)	0	6(5.5)
#BLOOD CREATINE PHOSPHOKINASE INCREASED	1(0.9)	0	1(0.9)	2(1.8)	1(0.9)	0	0	0	3(2.8)	2(1.8)	5(4.6)
BLOOD UREA INCREASED	2(1.8)	2(1.8)	0	1(0.9)	0	0	0	0	2(1.8)	3(2.8)	5(4.6)
LIPASE INCREASED	1(0.9)	0	1(0.9)	0	4(3.7)	0	0	0	5(4.6)	0	5(4.6)
#BLOOD ALKALINE PHOSPHATASE INCREASED	3(2.8)	0	0	0	0	0	0	0	3(2.8)	0	3(2.8)
BLOOD CREATININE INCREASED	1(0.9)	1(0.9)	0	1(0.9)	0	0	0	0	1(0.9)	2(1.8)	3(2.8)
BLOOD PRESSURE INCREASED	0	2(1.8)	0	0	0	0	0	0	0	2(1.8)	2(1.8)
#HEPATIC ENZYME INCREASED	2(1.8)	0	0	0	0	0	0	0	2(1.8)	0	2(1.8)

MedDRA: Medical Dictionary for Regulatory Activities. NR: Not related. R: Related. n = Number of subjects with at least one TEAE in each category (subjects with multiple AEs in each category are counted only once in each category). N = Total number of subjects in the relevant analysis population. % = Percentage of subjects with at least one TEAE in each category relative to the total number of subjects in the relevant analysis population. Treatment-emergent adverse events (TEAEs): Defined as AEs which started or worsened on or after the first study drug administration up to and including 14 days after the last study drug administration. Grade I,II,III, IV TEAEs: Defined as TEAEs for which the severity (DMID grade) is indicated as Grade 1 (mild), Grade 2 (moderate), Grade 3 (severe) and Grade 4 (potentially life-threatening) respectively. #: Indicates TEAEs of special interest. TEAEs of special interest: Identified by pre-specified SMQ codes as confirmed by TB Alliance . Adverse events: Coded using MedDRA Version 20.0.

Program: PRODUCTION\TABLES\PEL\T14 3 1 11 PEL.SAS (18JUN2019 12:32) File: T14 3 1 11 PEL.rtf

Table S4
Incidence of TEAEs by Severity and Relationship
Population: Safety

MedDRA System Organ Class<br>Preferred Term	Grade 1<br>Related	Grade 1<br>Not Related	Grade 2<br>Related	Grade 2<br>Not Related	Grade 3<br>Related	Grade 3<br>Not Related	Grade 4<br>Related	Grade 4<br>Not Related	Any<br>Related	Any<br>Not Related	Total<br>R+NR
INVESTIGATIONS											
WHITE BLOOD CELL COUNT INCREASED	0	1(0.9)	0	1(0.9)	0	0	0	0	0	2(1.8)	2(1.8)
ALBUMIN URINE PRESENT	1(0.9)	0	0	0	0	0	0	0	1(0.9)	0	1(0.9)
BLOOD CREATINE PHOSPHOKINASE MB INCREASED	0	0	1(0.9)	0	0	0	0	0	1(0.9)	0	1(0.9)
#BLOOD LACTIC ACID INCREASED	0	0	1(0.9)	0	0	0	0	0	1(0.9)	0	1(0.9)
BLOOD URIC ACID INCREASED	1(0.9)	0	0	0	0	0	0	0	1(0.9)	0	1(0.9)
CREATININE RENAL CLEARANCE DECREASED	0	0	1(0.9)	0	0	0	0	0	1(0.9)	0	1(0.9)
METABOLISM AND NUTRITION DISORDERS	23(21.1)	19(17.4)	15(13.8)	9(8.3)	4(3.7)	3(2.8)	2(1.8)	2(1.8)	33(30.3)	28(25.7)	51(46.8)
DECREASED APPETITE	17(15.6)	3(2.8)	6(5.5)	0	0	0	0	0	21(19.3)	3(2.8)	24(22.0)
HYPOGLYCAEMIA	2(1.8)	3(2.8)	1(0.9)	3(2.8)	1(0.9)	0	1(0.9)	2(1.8)	5(4.6)	7(6.4)	12(11.0)
ABNORMAL LOSS OF WEIGHT	3(2.8)	4(3.7)	3(2.8)	2(1.8)	0	1(0.9)	0	0	6(5.5)	7(6.4)	11(10.1)
HYPERAMYLASAEMIA	0	1(0.9)	3(2.8)	0	2(1.8)	0	0	0	5(4.6)	1(0.9)	6(5.5)
HYPOMAGNESAEMIA	0	4(3.7)	1(0.9)	0	0	0	0	0	1(0.9)	4(3.7)	5(4.6)
DEHYDRATION	1(0.9)	0	0	3(2.8)	0	0	0	0	1(0.9)	3(2.8)	4(3.7)
HYPERGLYCAEMIA	3(2.8)	2(1.8)	0	0	0	0	0	0	3(2.8)	2(1.8)	4(3.7)
HYPERKALAEMIA	0	1(0.9)	0	2(1.8)	0	1(0.9)	0	0	0	3(2.8)	3(2.8)
#HYPERLACTACIDAEMIA	1(0.9)	0	1(0.9)	1(0.9)	0	0	0	0	2(1.8)	1(0.9)	3(2.8)
#LACTIC ACIDOSIS	0	0	1(0.9)	0	1(0.9)	0	1(0.9)	0	3(2.8)	0	3(2.8)
HYPOCALCAEMIA	1(0.9)	1(0.9)	0	0	0	0	0	0	1(0.9)	1(0.9)	2(1.8)

MedDRA: Medical Dictionary for Regulatory Activities. NR: Not related. R: Related. n = Number of subjects with at least one TEAE in each category (subjects with multiple AEs in each category are counted only once in each category). N = Total number of subjects in the relevant analysis population. % = Percentage of subjects with at least one TEAE in each category relative to the total number of subjects in the relevant analysis population. Treatment-emergent adverse events (TEAEs): Defined as AEs which started or worsened on or after the first study drug administration up to and including 14 days after the last study drug administration. Grade I,II,III, IV TEAEs: Defined as TEAEs for which the severity (DMID grade) is indicated as Grade 1 (mild), Grade 2 (moderate), Grade 3 (severe) and Grade 4 (potentially life-threatening) respectively. #: Indicates TEAEs of special interest. TEAEs of special interest: Identified by pre-specified SMQ codes as confirmed by TB Alliance . Adverse events: Coded using MedDRA Version 20.0.

Program: PRODUCTION\TABLES\PEL\T14 3 1 11 PEL.SAS (18JUN2019 12:32) File: T14 3 1 11 PEL.rtf

Table S4
Incidence of TEAEs by Severity and Relationship
Population: Safety

MedDRA System Organ Class<br>Preferred Term	Grade 1<br>Related	Grade 1<br>Not Related	Grade 2<br>Related	Grade 2<br>Not Related	Grade 3<br>Related	Grade 3<br>Not Related	Grade 4<br>Related	Grade 4<br>Not Related	Any<br>Related	Any<br>Not Related	Total<br>R+NR
METABOLISM AND NUTRITION DISORDERS											
HYPOKALAEMIA	0	2(1.8)	0	0	0	0	0	0	0	2(1.8)	2(1.8)
HYPONATRAEMIA	0	2(1.8)	0	0	0	0	0	0	0	2(1.8)	2(1.8)
#ACIDOSIS	0	0	1(0.9)	0	0	0	0	0	1(0.9)	0	1(0.9)
DIABETES MELLITUS INADEQUATE CONTROL	0	0	0	0	0	1(0.9)	0	0	0	1(0.9)	1(0.9)
HYPERLIPASAEMIA	1(0.9)	0	0	0	0	0	0	0	1(0.9)	0	1(0.9)
HYPERURICAEMIA	0	1(0.9)	0	0	0	0	0	0	0	1(0.9)	1(0.9)
HYPOPHOSPHATAEMIA	0	1(0.9)	0	0	0	0	0	0	0	1(0.9)	1(0.9)
HYPOVOLAEMIA	1(0.9)	0	0	0	0	0	0	0	1(0.9)	0	1(0.9)
MUSCULOSKELETAL AND CONNECTIVE TISSUE DISORDERS	19(17.4)	19(17.4)	2(1.8)	3(2.8)	0	0	0	0	20(18.3)	22(20.2)	37(33.9)
BACK PAIN	3(2.8)	7(6.4)	1(0.9)	2(1.8)	0	0	0	0	4(3.7)	9(8.3)	13(11.9)
PAIN IN EXTREMITY	4(3.7)	3(2.8)	1(0.9)	0	0	0	0	0	5(4.6)	3(2.8)	8(7.3)
ARTHRALGIA	5(4.6)	2(1.8)	0	0	0	0	0	0	5(4.6)	2(1.8)	6(5.5)
COSTOCHONDRITIS	1(0.9)	5(4.6)	0	0	0	0	0	0	1(0.9)	5(4.6)	6(5.5)
#MYALGIA	3(2.8)	4(3.7)	0	0	0	0	0	0	3(2.8)	4(3.7)	6(5.5)
MUSCLE SPASMS	2(1.8)	2(1.8)	0	0	0	0	0	0	2(1.8)	2(1.8)	4(3.7)
FLANK PAIN	0	1(0.9)	0	1(0.9)	0	0	0	0	0	2(1.8)	2(1.8)
MUSCULOSKELETAL CHEST PAIN	1(0.9)	1(0.9)	0	0	0	0	0	0	1(0.9)	1(0.9)	2(1.8)
CHEST WALL MASS	0	1(0.9)	0	0	0	0	0	0	0	1(0.9)	1(0.9)

MedDRA: Medical Dictionary for Regulatory Activities. NR: Not related. R: Related. n = Number of subjects with at least one TEAE in each category (subjects with multiple AEs in each category are counted only once in each category). N = Total number of subjects in the relevant analysis population. % = Percentage of subjects with at least one TEAE in each category relative to the total number of subjects in the relevant analysis population. Treatment-emergent adverse events (TEAEs): Defined as AEs which started or worsened on or after the first study drug administration up to and including 14 days after the last study drug administration. Grade I,II,III, IV TEAEs: Defined as TEAEs for which the severity (DMID grade) is indicated as Grade 1 (mild), Grade 2 (moderate), Grade 3 (severe) and Grade 4 (potentially life-threatening) respectively. #: Indicates TEAEs of special interest. TEAEs of special interest: Identified by pre-specified SMQ codes as confirmed by TB Alliance . Adverse events: Coded using MedDRA Version 20.0.

Program: PRODUCTION\TABLES\PEL\T14 3 1 11 PEL.SAS (18JUN2019 12:32) File: T14 3 1 11 PEL.rtf

Table S4
Incidence of TEAEs by Severity and Relationship
Population: Safety

MedDRA System Organ Class<br>Preferred Term	Grade 1<br>Related	Grade 1<br>Not Related	Grade 2<br>Related	Grade 2<br>Not Related	Grade 3<br>Related	Grade 3<br>Not Related	Grade 4<br>Related	Grade 4<br>Not Related	Any<br>Related	Any<br>Not Related	Total<br>R+NR
MUSCULOSKELETAL AND CONNECTIVE TISSUE DISORDERS											
#MUSCULOSKELETAL PAIN	1(0.9)	0	0	0	0	0	0	0	1(0.9)	0	1(0.9)
MUSCULOSKELETAL STIFFNESS	1(0.9)	0	0	0	0	0	0	0	1(0.9)	0	1(0.9)
#MYALGIA INTERCOSTAL	0	1(0.9)	0	0	0	0	0	0	0	1(0.9)	1(0.9)
TEMPOROMANDIBULAR JOINT SYNDROME	1(0.9)	0	0	0	0	0	0	0	1(0.9)	0	1(0.9)
EYE DISORDERS	17(15.6)	13(11.9)	6(5.5)	1(0.9)	0	0	0	0	22(20.2)	14(12.8)	34(31.2)
#VISUAL ACUITY REDUCED	3(2.8)	1(0.9)	2(1.8)	0	0	0	0	0	5(4.6)	1(0.9)	6(5.5)
CONJUNCTIVITIS ALLERGIC	0	5(4.6)	0	0	0	0	0	0	0	5(4.6)	5(4.6)
EYE PAIN	3(2.8)	1(0.9)	1(0.9)	0	0	0	0	0	4(3.7)	1(0.9)	5(4.6)
EYE PRURITUS	1(0.9)	3(2.8)	0	0	0	0	0	0	1(0.9)	3(2.8)	4(3.7)
#VISUAL IMPAIRMENT	3(2.8)	0	1(0.9)	0	0	0	0	0	4(3.7)	0	4(3.7)
VISION BLURRED	3(2.8)	0	0	0	0	0	0	0	3(2.8)	0	3(2.8)
CONJUNCTIVAL HAEMORRHAGE	1(0.9)	1(0.9)	0	0	0	0	0	0	1(0.9)	1(0.9)	2(1.8)
DRY EYE	1(0.9)	1(0.9)	0	0	0	0	0	0	1(0.9)	1(0.9)	2(1.8)
EYE IRRITATION	2(1.8)	0	0	0	0	0	0	0	2(1.8)	0	2(1.8)
PTERYGIUM	0	2(1.8)	0	0	0	0	0	0	0	2(1.8)	2(1.8)
#AMBLYOPIA	0	0	0	1(0.9)	0	0	0	0	0	1(0.9)	1(0.9)
EYE SWELLING	1(0.9)	0	0	0	0	0	0	0	1(0.9)	0	1(0.9)
HYPERMETROPIA	0	1(0.9)	0	0	0	0	0	0	0	1(0.9)	1(0.9)

MedDRA: Medical Dictionary for Regulatory Activities. NR: Not related. R: Related. n = Number of subjects with at least one TEAE in each category (subjects with multiple AEs in each category are counted only once in each category). N = Total number of subjects in the relevant analysis population. % = Percentage of subjects with at least one TEAE in each category relative to the total number of subjects in the relevant analysis population. Treatment-emergent adverse events (TEAEs): Defined as AEs which started or worsened on or after the first study drug administration up to and including 14 days after the last study drug administration. Grade I,II,III, IV TEAEs: Defined as TEAEs for which the severity (DMID grade) is indicated as Grade 1 (mild), Grade 2 (moderate), Grade 3 (severe) and Grade 4 (potentially life-threatening) respectively. #: Indicates TEAEs of special interest. TEAEs of special interest: Identified by pre-specified SMQ codes as confirmed by TB Alliance . Adverse events: Coded using MedDRA Version 20.0.

Program: PRODUCTION\TABLES\PEL\T14_3_1_11_PEL.SAS (18JUN2019 12:32) File: T14_3_1_11_PEL.rtf

Table S4
Incidence of TEAEs by Severity and Relationship
Population: Safety

MedDRA System Organ Class	Grade 1		Grade 2		Grade 3		Grade 4		Any		Total
Preferred Term	Related	Not Related	Related	Not Related	Related	Not Related	Related	Not Related	Related	Not Related	R+NR
EYE DISORDERS											
IRIDOCYCLITIS	0	1(0.9)	0	0	0	0	0	0	0	1(0.9)	1(0.9)
LENS DISORDER	0	0	1(0.9)	0	0	0	0	0	1(0.9)	0	1(0.9)
MYOPIA	0	1(0.9)	0	0	0	0	0	0	0	1(0.9)	1(0.9)
#OPTIC DISC HYPERAEMIA	1(0.9)	0	0	0	0	0	0	0	1(0.9)	0	1(0.9)
#OPTIC NEUROPATHY	1(0.9)	0	0	0	0	0	0	0	1(0.9)	0	1(0.9)
#PAPILLOEDEMA	1(0.9)	0	0	0	0	0	0	0	1(0.9)	0	1(0.9)
PRESBYOPIA	0	0	1(0.9)	0	0	0	0	0	1(0.9)	0	1(0.9)
RETINAL HAEMORRHAGE	1(0.9)	0	0	0	0	0	0	0	1(0.9)	0	1(0.9)
GENERAL DISORDERS AND ADMINISTRATION SITE CONDITIONS	6(5.5)	10(9.2)	1(0.9)	2(1.8)	0	1(0.9)	1(0.9)	0	7(6.4)	13(11.9)	20(18.3)
OEDEMA PERIPHERAL	0	3(2.8)	0	1(0.9)	0	0	0	0	0	4(3.7)	4(3.7)
FATIGUE	3(2.8)	0	0	0	0	0	0	0	3(2.8)	0	3(2.8)
ASTHENIA	1(0.9)	0	1(0.9)	0	0	0	0	0	2(1.8)	0	2(1.8)
CHEST DISCOMFORT	0	1(0.9)	0	0	0	1(0.9)	0	0	0	2(1.8)	2(1.8)
NON-CARDIAC CHEST PAIN	0	1(0.9)	0	1(0.9)	0	0	0	0	0	2(1.8)	2(1.8)
PYREXIA	0	2(1.8)	0	0	0	0	0	0	0	2(1.8)	2(1.8)
CHEST PAIN	1(0.9)	0	0	0	0	0	0	0	1(0.9)	0	1(0.9)
MALAISE	1(0.9)	0	0	0	0	0	0	0	1(0.9)	0	1(0.9)
MULTIPLE ORGAN DYSFUNCTION SYNDROME	0	0	0	0	0	0	1(0.9)	0	1(0.9)	0	1(0.9)

MedDRA: Medical Dictionary for Regulatory Activities. NR: Not related. R: Related. n = Number of subjects with at least one TEAE in each category (subjects with multiple AEs in each category are counted only once in each category). N = Total number of subjects in the relevant analysis population. % = Percentage of subjects with at least one TEAE in each category relative to the total number of subjects in the relevant analysis population. Treatment-emergent adverse events (TEAEs): Defined as AEs which started or worsened on or after the first study drug administration up to and including 14 days after the last study drug administration. Grade I,II,III, IV TEAEs: Defined as TEAEs for which the severity (DMID grade) is indicated as Grade 1 (mild), Grade 2 (moderate), Grade 3 (severe) and Grade 4 (potentially life-threatening) respectively. #: Indicates TEAEs of special interest. TEAEs of special interest: Identified by pre-specified SMQ codes as confirmed by TB Alliance . Adverse events: Coded using MedDRA Version 20.0.

Program: PRODUCTION\TABLES\PEL\T14 3 1 11 PEL.SAS (18JUN2019 12:32) File: T14 3 1 11 PEL.rtf

Table S4
Incidence of TEAEs by Severity and Relationship
Population: Safety

MedDRA System Organ Class<br>Preferred Term	Grade 1<br>Related	Grade 1<br>Not Related	Grade 2<br>Related	Grade 2<br>Not Related	Grade 3<br>Related	Grade 3<br>Not Related	Grade 4<br>Related	Grade 4<br>Not Related	Any<br>Related	Any<br>Not Related	Total<br>R+NR
GENERAL DISORDERS AND ADMINISTRATION SITE CONDITIONS											
THIRST	0	1(0.9)	0	0	0	0	0	0	0	1(0.9)	1(0.9)
VESSEL PUNCTURE SITE PAIN	0	1(0.9)	0	0	0	0	0	0	0	1(0.9)	1(0.9)
XEROSIS	0	1(0.9)	0	0	0	0	0	0	0	1(0.9)	1(0.9)
REPRODUCTIVE SYSTEM AND BREAST DISORDERS	3(2.8)	13(11.9)	1(0.9)	0	0	0	0	0	4(3.7)	13(11.9)	15(13.8)
VAGINAL DISCHARGE	0	4(3.7)	1(0.9)	0	0	0	0	0	1(0.9)	4(3.7)	4(3.7)
METRORRHAGIA	1(0.9)	2(1.8)	0	0	0	0	0	0	1(0.9)	2(1.8)	3(2.8)
ERECTILE DYSFUNCTION	2(1.8)	0	0	0	0	0	0	0	2(1.8)	0	2(1.8)
MENORRHAGIA	0	2(1.8)	0	0	0	0	0	0	0	2(1.8)	2(1.8)
VAGINAL HAEMORRHAGE	0	2(1.8)	0	0	0	0	0	0	0	2(1.8)	2(1.8)
GALACTORRHOEA	0	1(0.9)	0	0	0	0	0	0	0	1(0.9)	1(0.9)
GENITAL RASH	0	1(0.9)	0	0	0	0	0	0	0	1(0.9)	1(0.9)
GENITAL ULCERATION	0	1(0.9)	0	0	0	0	0	0	0	1(0.9)	1(0.9)
PERINEAL PAIN	0	1(0.9)	0	0	0	0	0	0	0	1(0.9)	1(0.9)
VASCULAR DISORDERS	0	6(5.5)	0	7(6.4)	1(0.9)	0	0	0	1(0.9)	11(10.1)	12(11.0)
HYPERTENSION	0	3(2.8)	0	5(4.6)	0	0	0	0	0	8(7.3)	8(7.3)
HYPOTENSION	0	1(0.9)	0	1(0.9)	1(0.9)	0	0	0	1(0.9)	1(0.9)	2(1.8)
DEEP VEIN THROMBOSIS	0	0	0	1(0.9)	0	0	0	0	0	1(0.9)	1(0.9)
FLUSHING	0	1(0.9)	0	0	0	0	0	0	0	1(0.9)	1(0.9)

MedDRA: Medical Dictionary for Regulatory Activities. NR: Not related. R: Related. n = Number of subjects with at least one TEAE in each category (subjects with multiple AEs in each category are counted only once in each category). N = Total number of subjects in the relevant analysis population. % = Percentage of subjects with at least one TEAE in each category relative to the total number of subjects in the relevant analysis population. Treatment-emergent adverse events (TEAEs): Defined as AEs which started or worsened on or after the first study drug administration up to and including 14 days after the last study drug administration. Grade I,II,III, IV TEAEs: Defined as TEAEs for which the severity (DMID grade) is indicated as Grade 1 (mild), Grade 2 (moderate), Grade 3 (severe) and Grade 4 (potentially life-threatening) respectively. #: Indicates TEAEs of special interest. TEAEs of special interest: Identified by pre-specified SMQ codes as confirmed by TB Alliance . Adverse events: Coded using MedDRA Version 20.0.

Program: PRODUCTION\TABLES\PEL\T14 3 1 11 PEL.SAS (18JUN2019 12:32) File: T14 3 1 11 PEL.rtf

Table S4
Incidence of TEAEs by Severity and Relationship
Population: Safety

MedDRA System Organ Class<br>Preferred Term	Grade 1<br>Related	Grade 1<br>Not Related	Grade 2<br>Related	Grade 2<br>Not Related	Grade 3<br>Related	Grade 3<br>Not Related	Grade 4<br>Related	Grade 4<br>Not Related	Any<br>Related	Any<br>Not Related	Total<br>R+NR
VASCULAR DISORDERS											
HAEMATOMA	0	1(0.9)	0	0	0	0	0	0	0	1(0.9)	1(0.9)
PERIPHERAL COLDNESS	0	1(0.9)	0	0	0	0	0	0	0	1(0.9)	1(0.9)
CARDIAC DISORDERS	4(3.7)	3(2.8)	1(0.9)	2(1.8)	0	0	0	0	5(4.6)	5(4.6)	10(9.2)
#PALPITATIONS	1(0.9)	1(0.9)	1(0.9)	0	0	0	0	0	2(1.8)	1(0.9)	3(2.8)
ANGINA PECTORIS	1(0.9)	0	0	1(0.9)	0	0	0	0	1(0.9)	1(0.9)	2(1.8)
#BRADYCARDIA	0	1(0.9)	0	0	0	0	0	0	0	1(0.9)	1(0.9)
CARDIAC FAILURE	0	1(0.9)	0	0	0	0	0	0	0	1(0.9)	1(0.9)
COR PULMONALE	0	0	0	1(0.9)	0	0	0	0	0	1(0.9)	1(0.9)
#SINUS BRADYCARDIA	0	0	0	1(0.9)	0	0	0	0	0	1(0.9)	1(0.9)
#SINUS TACHYCARDIA	1(0.9)	0	0	0	0	0	0	0	1(0.9)	0	1(0.9)
#TACHYCARDIA	1(0.9)	0	0	0	0	0	0	0	1(0.9)	0	1(0.9)
EAR AND LABYRINTH DISORDERS	1(0.9)	6(5.5)	0	4(3.7)	0	0	0	0	1(0.9)	9(8.3)	10(9.2)
EAR PAIN	0	2(1.8)	0	1(0.9)	0	0	0	0	0	3(2.8)	3(2.8)
TYMPANIC MEMBRANE PERFORATION	0	2(1.8)	0	1(0.9)	0	0	0	0	0	3(2.8)	3(2.8)
EXCESSIVE CERUMEN PRODUCTION	0	1(0.9)	0	1(0.9)	0	0	0	0	0	2(1.8)	2(1.8)
AURICULAR SWELLING	0	1(0.9)	0	0	0	0	0	0	0	1(0.9)	1(0.9)
CERUMEN IMPACTION	0	1(0.9)	0	0	0	0	0	0	0	1(0.9)	1(0.9)
DEAFNESS	1(0.9)	0	0	0	0	0	0	0	1(0.9)	0	1(0.9)

MedDRA: Medical Dictionary for Regulatory Activities. NR: Not related. R: Related. n = Number of subjects with at least one TEAE in each category (subjects with multiple AEs in each category are counted only once in each category). N = Total number of subjects in the relevant analysis population. % = Percentage of subjects with at least one TEAE in each category relative to the total number of subjects in the relevant analysis population. Treatment-emergent adverse events (TEAEs): Defined as AEs which started or worsened on or after the first study drug administration up to and including 14 days after the last study drug administration. Grade I,II,III, IV TEAEs: Defined as TEAEs for which the severity (DMID grade) is indicated as Grade 1 (mild), Grade 2 (moderate), Grade 3 (severe) and Grade 4 (potentially life-threatening) respectively. #: Indicates TEAEs of special interest. TEAEs of special interest: Identified by pre-specified SMQ codes as confirmed by TB Alliance . Adverse events: Coded using MedDRA Version 20.0.

Program: PRODUCTION\TABLES\PEL\T14_3_1_11_PEL.SAS (18JUN2019 12:32) File: T14_3_1_11_PEL.rtf

Table S4
Incidence of TEAEs by Severity and Relationship
Population: Safety

	Grade 1		Grade 2		Grade 3		Grade 4		Any		Total
MedDRA System Organ Class<br>Preferred Term	Related	Not Related	Related	Not Related	Related	Not Related	Related	Not Related	Related	Not Related	R+NR
EAR AND LABYRINTH DISORDERS											
EAR PRURITUS	0	1(0.9)	0	0	0	0	0	0	0	1(0.9)	1(0.9)
MASTOID EFFUSION	0	0	0	1(0.9)	0	0	0	0	0	1(0.9)	1(0.9)
INJURY, POISONING AND PROCEDURAL COMPLICATIONS	0	7(6.4)	0	3(2.8)	0	0	0	0	0	10(9.2)	10(9.2)
ARTHROPOD BITE	0	3(2.8)	0	1(0.9)	0	0	0	0	0	4(3.7)	4(3.7)
GINGIVAL INJURY	0	1(0.9)	0	0	0	0	0	0	0	1(0.9)	1(0.9)
INCISION SITE PAIN	0	0	0	1(0.9)	0	0	0	0	0	1(0.9)	1(0.9)
LACERATION	0	0	0	1(0.9)	0	0	0	0	0	1(0.9)	1(0.9)
LIGAMENT SPRAIN	0	1(0.9)	0	0	0	0	0	0	0	1(0.9)	1(0.9)
POST-TRAUMATIC PAIN	0	1(0.9)	0	0	0	0	0	0	0	1(0.9)	1(0.9)
SKIN ABRASION	0	1(0.9)	0	0	0	0	0	0	0	1(0.9)	1(0.9)
SOFT TISSUE INJURY	0	1(0.9)	0	0	0	0	0	0	0	1(0.9)	1(0.9)
PSYCHIATRIC DISORDERS	3(2.8)	5(4.6)	2(1.8)	3(2.8)	0	0	0	1(0.9)	4(3.7)	7(6.4)	10(9.2)
INSOMNIA	2(1.8)	3(2.8)	1(0.9)	1(0.9)	0	0	0	0	3(2.8)	4(3.7)	6(5.5)
ANXIETY	1(0.9)	1(0.9)	0	0	0	0	0	0	1(0.9)	1(0.9)	2(1.8)
DEPRESSION	0	0	1(0.9)	1(0.9)	0	0	0	0	1(0.9)	1(0.9)	2(1.8)
DEPRESSION SUICIDAL	0	0	0	0	0	0	0	1(0.9)	0	1(0.9)	1(0.9)
GENERALISED ANXIETY DISORDER	0	1(0.9)	0	1(0.9)	0	0	0	0	0	1(0.9)	1(0.9)
MAJOR DEPRESSION	0	1(0.9)	0	0	0	0	0	0	0	1(0.9)	1(0.9)

MedDRA: Medical Dictionary for Regulatory Activities. NR: Not related. R: Related. n = Number of subjects with at least one TEAE in each category (subjects with multiple AEs in each category are counted only once in each category). N = Total number of subjects in the relevant analysis population. % = Percentage of subjects with at least one TEAE in each category relative to the total number of subjects in the relevant analysis population. Treatment-emergent adverse events (TEAEs): Defined as AEs which started or worsened on or after the first study drug administration up to and including 14 days after the last study drug administration. Grade I,II,III, IV TEAEs: Defined as TEAEs for which the severity (DMID grade) is indicated as Grade 1 (mild), Grade 2 (moderate), Grade 3 (severe) and Grade 4 (potentially life-threatening) respectively. #: Indicates TEAEs of special interest. TEAEs of special interest: Identified by pre-specified SMQ codes as confirmed by TB Alliance . Adverse events: Coded using MedDRA Version 20.0.

Program: PRODUCTION\TABLES\PEL\T14_3_1_11_PEL.SAS (18JUN2019 12:32) File: T14_3_1_11_PEL.rtf

Table S4
Incidence of TEAEs by Severity and Relationship
Population: Safety

MedDRA System Organ Class<br>Preferred Term	Grade 1<br>Related	Grade 1<br>Not Related	Grade 2<br>Related	Grade 2<br>Not Related	Grade 3<br>Related	Grade 3<br>Not Related	Grade 4<br>Related	Grade 4<br>Not Related	Any<br>Related	Any<br>Not Related	Total<br>R+NR
HEPATOBILIARY DISORDERS	2(1.8)	0	3(2.8)	1(0.9)	1(0.9)	0	1(0.9)	0	7(6.4)	1(0.9)	8(7.3)
#DRUG-INDUCED LIVER INJURY	0	0	1(0.9)	0	1(0.9)	0	0	0	2(1.8)	0	2(1.8)
#HYPERBILIRUBINAEMIA	0	0	1(0.9)	0	0	0	1(0.9)	0	2(1.8)	0	2(1.8)
BILE DUCT STONE	0	0	0	1(0.9)	0	0	0	0	0	1(0.9)	1(0.9)
#HEPATIC FUNCTION ABNORMAL	0	0	1(0.9)	0	0	0	0	0	1(0.9)	0	1(0.9)
#HEPATOMEGALY	1(0.9)	0	0	0	0	0	0	0	1(0.9)	0	1(0.9)
#JAUNDICE	1(0.9)	0	0	0	0	0	0	0	1(0.9)	0	1(0.9)
RENAL AND URINARY DISORDERS	0	3(2.8)	0	1(0.9)	0	0	0	0	0	4(3.7)	4(3.7)
AZOTAEMIA	0	1(0.9)	0	0	0	0	0	0	0	1(0.9)	1(0.9)
POLLAKIURIA	0	1(0.9)	0	0	0	0	0	0	0	1(0.9)	1(0.9)
PROTEINURIA	0	0	0	1(0.9)	0	0	0	0	0	1(0.9)	1(0.9)
RENAL FAILURE	0	1(0.9)	0	0	0	0	0	0	0	1(0.9)	1(0.9)
IMMUNE SYSTEM DISORDERS	0	1(0.9)	0	0	0	0	0	0	0	1(0.9)	1(0.9)
SEASONAL ALLERGY	0	1(0.9)	0	0	0	0	0	0	0	1(0.9)	1(0.9)

MedDRA: Medical Dictionary for Regulatory Activities. NR: Not related. R: Related. n = Number of subjects with at least one TEAE in each category (subjects with multiple AEs in each category are counted only once in each category). N = Total number of subjects in the relevant analysis population. % = Percentage of subjects with at least one TEAE in each category relative to the total number of subjects in the relevant analysis population. Treatment-emergent adverse events (TEAEs): Defined as AEs which started or worsened on or after the first study drug administration up to and including 14 days after the last study drug administration. Grade I,II,III, IV TEAEs: Defined as TEAEs for which the severity (DMID grade) is indicated as Grade 1 (mild), Grade 2 (moderate), Grade 3 (severe) and Grade 4 (potentially life-threatening) respectively. #: Indicates TEAEs of special interest. TEAEs of special interest: Identified by pre-specified SMQ codes as confirmed by TB Alliance . Adverse events: Coded using MedDRA Version 20.0.

Program: PRODUCTION\TABLES\PEL\T14_3_1_11_PEL.SAS (18JUN2019 12:32) File: T14_3_1_11_PEL.rtf

Table S5 Serious adverse events by system organ class and preferred term

Table S5
Incidence of Serious TEAEs (Including Death)
Population: Safety

MedDRA System Organ Class<br>Preferred Term	Statistic	B-L-Pa<br>(N=109)	
SUBJECTS WITH AT LEAST ONE SERIOUS TEAE	n (%) E	19 (17.4)	36
INFECTIONS AND INFESTATIONS	n (%) E	7 (6.4)	11
PNEUMONIA	n (%) E	3 (2.8)	3
PULMONARY TUBERCULOSIS	n (%) E	3 (2.8)	3
SEPSIS	n (%) E	2 (1.8)	2
DISSEMINATED TUBERCULOSIS	n (%) E	1 (0.9)	1
SEPTIC SHOCK	n (%) E	1 (0.9)	1
TUBERCULOMA OF CENTRAL NERVOUS SYSTEM	n (%) E	1 (0.9)	1
GASTROINTESTINAL DISORDERS	n (%) E	5 (4.6)	5
ABDOMINAL PAIN UPPER	n (%) E	1 (0.9)	1
HAEMATEMESIS	n (%) E	1 (0.9)	1
#PANCREATITIS	n (%) E	1 (0.9)	1
#PANCREATITIS HAEMORRHAGIC	n (%) E	1 (0.9)	1
UPPER GASTROINTESTINAL HAEMORRHAGE	n (%) E	1 (0.9)	1

MedDRA: Medical Dictionary for Regulatory Activities. n = Number of subjects with at least one TEAE in each category (subjects with multiple AEs in each category are counted only once in each category). E = Number of cases in each category (events). N = Total number of subjects in the relevant analysis population. % = Percentage of subjects with at least one TEAE in each category relative to the total number of subjects in the relevant analysis population. Treatment-emergent adverse events (TEAEs): Defined as AEs which started or worsened on or after the first study drug administration up to and including 14 days after the last study drug administration. TEAEs leading to death: Defined as TEAEs for which outcome is indicated as fatal. Serious TEAEs: Defined as TEAEs for which serious is indicated as yes. #: Indicates TEAEs of special interest. TEAEs of special interest: Identified by pre-specified SMQ codes as confirmed by TB Alliance. Adverse events: Coded using MedDRA Version 20.0.

Program: PRODUCTION\TABLES\MANUSCRIPT\T14 3 1 4.SAS (05JUL2019 05:48) File: T14 3 1 4 MANST.rtf

Table S5
Incidence of Serious TEAEs (Including Death)
Population: Safety

MedDRA System Organ Class<br>Preferred Term	Statistic	B-L-Pa (N=109)
METABOLISM AND NUTRITION DISORDERS	n (%) E	4 (3.7) 4
HYPOGLYCAEMIA	n (%) E	2 (1.8) 2
ABNORMAL LOSS OF WEIGHT	n (%) E	1 (0.9) 1
#LACTIC ACIDOSIS	n (%) E	1 (0.9) 1
NERVOUS SYSTEM DISORDERS	n (%) E	4 (3.7) 4
#GENERALISED TONIC-CLONIC SEIZURE	n (%) E	1 (0.9) 1
#OPTIC NEURITIS	n (%) E	1 (0.9) 1
#SEIZURE	n (%) E	1 (0.9) 1
#SYNCOPE	n (%) E	1 (0.9) 1
BLOOD AND LYMPHATIC SYSTEM DISORDERS	n (%) E	3 (2.8) 3
#ANAEMIA	n (%) E	2 (1.8) 2
#NEUTROPENIA	n (%) E	1 (0.9) 1
RESPIRATORY, THORACIC AND MEDIASTINAL DISORDERS	n (%) E	3 (2.8) 4
ASTHMA	n (%) E	1 (0.9) 1
DYSPNOEA	n (%) E	1 (0.9) 1
HAEMOPTYSIS	n (%) E	1 (0.9) 1

MedDRA: Medical Dictionary for Regulatory Activities. n = Number of subjects with at least one TEAE in each category (subjects with multiple AEs in each category are counted only once in each category). E = Number of cases in each category (events). N = Total number of subjects in the relevant analysis population. % = Percentage of subjects with at least one TEAE in each category relative to the total number of subjects in the relevant analysis population. Treatment-emergent adverse events (TEAEs): Defined as AEs which started or worsened on or after the first study drug administration up to and including 14 days after the last study drug administration. TEAEs leading to death: Defined as TEAEs for which outcome is indicated as fatal. Serious TEAEs: Defined as TEAEs for which serious is indicated as yes. #: Indicates TEAEs of special interest. TEAEs of special interest: Identified by pre-specified SMQ codes as confirmed by TB Alliance. Adverse events: Coded using MedDRA Version 20.0.

Program: PRODUCTION\TABLES\MANUSCRIPT\T14_3_1_4.SAS (05JUL2019 05:48) File: T14_3_1_4_MANST.rtf

Table S5
Incidence of Serious TEAEs (Including Death)
Population: Safety

MedDRA System Organ Class<br>Preferred Term	Statistic	B-L-Pa<br>(N=109)
RESPIRATORY, THORACIC AND MEDIASTINAL DISORDERS		
PNEUMOTHORAX SPONTANEOUS	n (%) E	1 (0.9) 1
PSYCHIATRIC DISORDERS	n (%) E	2 (1.8) 2
DEPRESSION SUICIDAL	n (%) E	1 (0.9) 1
GENERALISED ANXIETY DISORDER	n (%) E	1 (0.9) 1
EYE DISORDERS	n (%) E	1 (0.9) 1
#OPTIC NEUROPATHY	n (%) E	1 (0.9) 1
GENERAL DISORDERS AND ADMINISTRATION SITE CONDITIONS	n (%) E	1 (0.9) 1
MULTIPLE ORGAN DYSFUNCTION SYNDROME	n (%) E	1 (0.9) 1
INVESTIGATIONS	n (%) E	1 (0.9) 1
#TRANSAMINASES INCREASED	n (%) E	1 (0.9) 1

MedDRA: Medical Dictionary for Regulatory Activities. n = Number of subjects with at least one TEAE in each category (subjects with multiple AEs in each category are counted only once in each category). E = Number of cases in each category (events). N = Total number of subjects in the relevant analysis population. % = Percentage of subjects with at least one TEAE in each category relative to the total number of subjects in the relevant analysis population. Treatment-emergent adverse events (TEAEs): Defined as AEs which started or worsened on or after the first study drug administration up to and including 14 days after the last study drug administration. TEAEs leading to death: Defined as TEAEs for which outcome is indicated as fatal. Serious TEAEs: Defined as TEAEs for which serious is indicated as yes. #: Indicates TEAEs of special interest. TEAEs of special interest: Identified by pre-specified SMQ codes as confirmed by TB Alliance. Adverse events: Coded using MedDRA Version 20.0.

Program: PRODUCTION\TABLES\MANUSCRIPT\T14 3 1 4.SAS (05JUL2019 05:48) File: T14 3 1 4 MANST.rtf

Figure S1 Time to first reduction or interruption of linezolid

i) Due to Peripheral neuropathy; ii) Due to Myelosuppression; A Overall; B by TB Type;

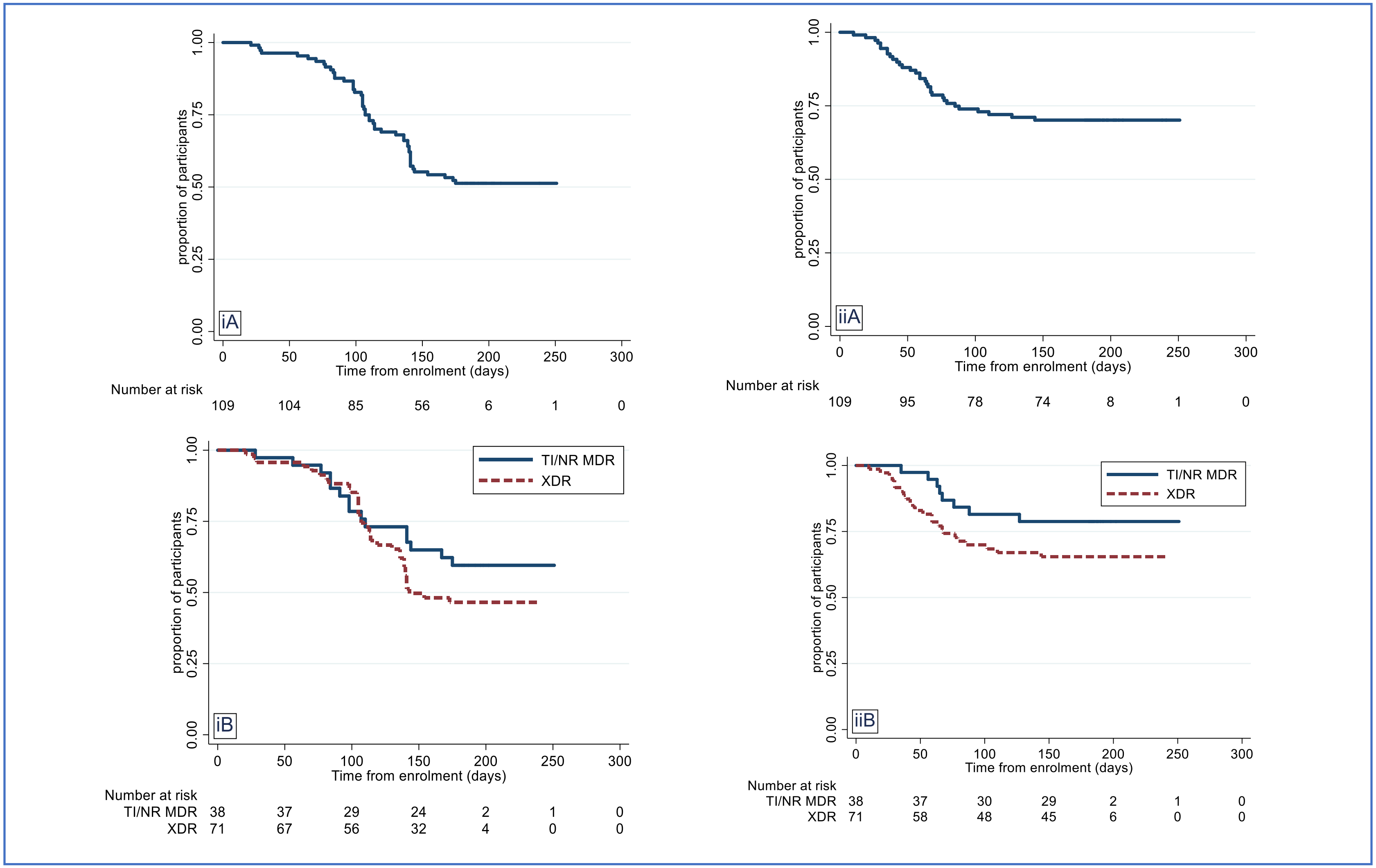

Supplement: Supplementary file 1 [file NEJM-2020-1901814-s1.pdf]
